# Experimental and theoretical investigation of parameter-driven stability and chaos in a non-linear neuron circuit model

**DOI:** 10.1371/journal.pone.0360729

**Published:** 2026-11-06

**Authors:** Haiqa Ehsan, Haseeba Sajjad, Adil Jhangeer, Lubomír Říha

**Affiliations:** 1 IT4Innovations, VSB-Technical University of Ostrava, Ostrava-Poruba, Czech Republic; 2 Faculty of Electrical Engineering and Computer Science, VSB-Technical University of Ostrava, Ostrava-Poruba, Czech Republic; 3 Center for Theoretical Physics, Khazar University, Baku, Azerbaijan, Azerbaijan; 4 Department of Computer Engineering, Biruni University, Istanbul, Turkey; Lanzhou University of Technology, CHINA

## Abstract

In this study, a neural-inspired electronic circuit consisting of a pressure-sensitive FitzHugh-Nagumo model with the addition of a piezoelectric bi-membrane and two capacitors is explored. The governing equations are obtained analytically from Kirchhoff’s laws, which represent the nonlinear interactions, oscillatory response, and stability of the circuit. A perturbation term is added to investigate the deviations from the system potential, and the transitions in the system are studied under the assumption of the model. By applying statistical methods and eigenvalue analysis, the parameter space is systematically subdivided and examined, and the results show that the behavior of the system is dominated by divergent behavior, although some periodic oscillatory behavior is seen in certain regions. This highlights the importance of initial conditions and parameter selection on the behavior of the system, which are essential to obtaining stable behavior and developing an efficient control design. Furthermore, an ensemble-size sensitivity analysis is performed to verify the robustness and convergence of the statistical characterization across different numbers of initial conditions. Phase disturbance plots, power spectrum density plots, attractor reconstruction plots, return maps, and noise detection maps are used to analyze the system dynamics. In addition, a multi-sum (multi-parameter interaction) diagram is created to depict the interactions between circuit variables and the overall system behavior.

## Introduction

The investigation of the dynamical behavior of neural circuits is a fundamental research area in both neuroscience and engineering [1,2]. The human nervous system has the ability to encode and represent information, which is a hallmark characteristic of humans. Neuron-like circuits have also been used in the field of electronics for complicated modeling of systems, control, and signal processing. Applications of technologies such as brain-machine interfaces (brain-computer interfaces), neural interfaces, and neuro-prosthetics are similarly contingent upon the ability to manipulate and regulate neuronal responses [3–5]. Investigating intricate patterns of electrical activity induction and mechanisms of neuronal cooperation from a purely experimental angle is not trivial [6]. These circuits simulate the electrical properties of actual nerves by limiting feedback with resistors, damping with capacitors, and regulating current with inductors. The presence of non-linear components enables the system to exhibit oscillations and spiking behavior by incorporating threshold dynamics. The external stimulation and ion-channel regulation of neuronal electrical behavior are now highlighted as essential factors by recent advances in the field of nonlinear dynamics of neurons. Yang et al. [7] investigated the electrical activity of functional neurons under various external stimuli, and showed that a variety of external excitations can lead to transitions between various states of the neuron, including resting, periodic firing, bursting and chaotic oscillatory states. Further, adaptive regulation of ion channels has been shown to be a major factor in tuning the electrical activity of the neurons under various physiological conditions and hence control their dynamic stability and excitability [8]. These studies demonstrate that external excitation and adaptive regulatory mechanisms play fundamental roles in shaping nonlinear neuronal dynamics. Motivated by these developments, the present work investigates a piezoelectric bi-membrane neuron model capable of exhibiting rich nonlinear behaviors under external forcing and parameter variations. In recent years, it has been shown that bi-membrane neuron-like circuits can display a variety of nonlinear dynamics, depending on the external stimuli and the coupling method. For instance, in the study of bursting synchronization and excitability, electromagnetic stimulation has been studied, and it is found that the external stimulus has a great effect on the activity of the neurons. The bursting dynamics have also been studied in modified bi-membrane neuron-like circuits composed of resistive switching and memristive components, where the nonlinear coupling between the components has been shown to play a crucial role in the determination of the behavior of the neurons. Furthermore, memristive current arrays with neurons with two membranes have been studied, and complex discrete images including disordered spiral waves have been observed, that might be used for image encryption. These findings highlight the beautiful dynamic phenomena of bi-membrane neuron-like systems and encourage more studies of the systems responses to various types of coupling and external excitation. In this context, the present work concentrates on the study of the nonlinear dynamics of the piezoelectric bi-membrane neuron-like circuit and its response under external excitations and variations of the circuit parameters. In recent decades, researchers have made it possible to better understand signal transmission within the nervous system by developing several models of nonlinear neurons based on clinical electro-physiological tests utilizing these sophisticated approaches. The importance of bi-capacitors added to neuron circuits is gaining attention in this research [9,10], since they can modify parameters such as conductance and input current gain to greatly affect system dynamics. The physical characteristics of the membrane also vary when the devices linked to the capacitors are different, such as resistors, piezoelectric materials, thermistors, or memristors. Two capacitors are needed from the point of view of circuit representation. Understanding these problems helps designers create functional neural circuits. Our understanding of the complex dynamics of the nervous system has been greatly improved by this multidisciplinary approach that draws upon experimental data, mathematical modeling, and technological development [11,12].

Recent studies have further investigated the electrical implementation and externally driven dynamics of nonlinear neuron circuits. Binczak et al. [13] experimentally investigated an electrical FHN neuron and characterized different dynamical regimes, including excitation thresholds, bistability, and oscillations. In addition, piezoelectric elements have been incorporated into functional neural circuits to reproduce and investigate stimulus-dependent neuronal dynamics [14]. Very recent work by Yang et al. [15] investigated the electrical activity of a functional neuron under diverse external stimuli and demonstrated that changes in the type and intensity of external stimulation can induce different neuronal activity patterns. These studies demonstrate the importance of external excitation and circuit elements in regulating nonlinear neuronal dynamics. However, a systematic characterization of the state-space response of a piezoelectric FHN electrical circuit over different excitation frequencies and voltage amplitudes remains insufficiently explored. Therefore, the present study focuses on the dynamical behavior of the proposed electrical FHN circuit under systematically varied excitation conditions, with particular emphasis on the resulting phase portraits and changes in the circuit state trajectories.

The membrane potential of existing neurons is usually represented by a single capacitive variable, which ignores the characteristics of the neuronal membrane, especially the physical characteristics of the intracellular medium and the distinctions between the interior and exterior of the membrane [16]. By scaling the variables and parameters of the circuit equations, the dimensionless neuron model and the energy function are obtained. As a result, changes in electromagnetic fields across the membrane and the influence of the intracellular medium on neuronal electrical activity can be represented using two separate capacitive variables. We specifically suggest a pressure-sensitive FitzHugh-Nagumo neuron (FHN) model that includes two parallel capacitors spaced apart by piezoelectric materials [17]. Excitable systems, including neurons, behave according to a simplified mathematical model called the FHN model. The dynamic characteristics of the neuron circuits are extremely sensitive to changes in the elasticity of embedded materials, as previous research has shown [18]. In order to better imitate neural functions and lower circuit power consumption, capacitive neural networks offer a novel physical embodiment of neural networks. This neuron circuit exploits the relationship between the electrical and mechanical domains for advanced computing and sensing technologies and may find use in bio-inspired signal processing, smart sensor networks, and brain-computer interfaces. In addition to reducing circuit power consumption, capacitive neural networks offer a novel physical embodiment of neural networks that more accurately imitate neural processes.

For the purpose of improving functionality, especially in modeling auditory processes [19], emphasizes the incorporation of physical devices, such as piezoelectric materials, into neural networks. It is well known that recent developments in semi-analytical and numerical techniques, including the time-domain minimal residual method, are effective for examining the dynamics of neural systems. The potential of mem-capacitors to mimic biological synaptic behavior is described in [20], thus enhancing information processing capacities in electronic systems, and emphasizes the importance of the Miller effect in altering circuit properties, especially in amplifiers. In [21] alternative neuron models and multiplier-free solutions are covered, along with the drawbacks of the original dependence of the FHN model on analog multipliers. Numerous studies have been conducted on the remarkable physical phenomena of chaos [22]. The neuronal circuit model can be employed as a chaotic system since it is well known that the brain functions in a chaotic state and that nonlinear dynamics in the neural membrane can cause chaos in neurons and related bifurcations [23]. Hyperchaos, multistability, and hidden attractors are examples of rich dynamic behaviors found in chaotic circuits with memory components [24].

Each parameter affects the stability, damping, and oscillatory behavior of an electrical system, influencing both its transient and steady-state response. Increases in the values of *C*_1_ and *C*_2_ will result in a slower and more stable response, whereas decreases in these values would result in a faster response and potential instability. Likewise, non-linearity and predictability are affected by series resistance $R_{S}$. Smoother oscillations and a more stable response are guaranteed when the non-linearity is reduced by a higher $R_{S}$. On the other hand, chaotic behavior and unpredictable oscillations may result from increased nonlinearity caused by a lower $R_{S}$. In general, damping is influenced by general resistance *R*. Stronger damping occurs in the system as *R* is raised, suppressing oscillations, and stabilizing the response. Reducing *R* results in less damping, which causes longer and more intense oscillations that can compromise stability. The inductance of the system *L* affects the rate of change of the current. Due to energy storage effects, a larger *L* results in a slower and more regulated response, avoiding sharp fluctuations. A faster reaction is possible with a lower *L*, although instability may be introduced due to high-frequency oscillations. The strength of the oscillations is directly affected by the supply voltage $V_{S}$. Because the system absorbs more energy when $V_{S}$ increases, the oscillations become more noticeable and may enhance the temporary effects. On the other hand, lowering $V_{S}$ results in a constant, non-oscillatory condition by weakening the response and, if done excessively, stopping oscillations entirely.

Systematic parameter space categorization evaluates dynamic system behaviors by systemically changing parameters, thus assessing their behavior patterns. The core elements of this method consist of stability oscillations and performance output outcomes that assess separate parameter configurations. Different parameter conditions drive the systematic eigenvalue analysis toward stable oscillatory or unstable systemic responses. As a fundamental investigative tool, this method allows experts to track the performance of the control system together with parameter-induced stability changes that lead to system optimization. Engineers can rely on this method to enable stability assurance through specific adjustments, which results in predictable and reliable operations. Engineers and controllers rely on this approach because it remains a standard practice in maintaining stability and optimizing control system performance. This method enables stabilization and performance improvement in mechanical systems, electrical circuits, robotic systems, and aerospace engineering applications. Nonlinear ordinary differential equations (ODEs) are essential modeling tools for understanding complex dynamic systems in many scientific disciplines. Two factors create analysis challenges for these systems and their classification procedure because they produce various styles of system dynamics through parameter interactions.

Modern computational methods, mathematical analysis methods, and machine learning approaches enable scientists to perform organized domain categorizations of parameter space. Scientists use suitable subspaces to categorize their parameter domains in ODEs with non-linearity. The category derives its definition from the acquisition of knowledge about stability patterns, oscillation ranges, and chaotic behavior domains. System dynamics predictions need accurate results from complete parameter understanding during multistage assessments of different conditions. Recent research on systematic methods that classify parameter domains in non-linear ODE systems receives a thorough review along with their core approaches and practical applications and potential future research directions.

Systematic techniques seek to analyze parameter space distributions, and researchers have raised such demands over the past few years. In [25], MOEA parameter identification combined with ANNs separates parameters during dynamic behavior zones. This paper approach accomplishes the systematic classification of parameter domains regarding admissibility for non-linear ODE. Parameter space investigations help define symbolic and formal series techniques for integrability and symmetry of ODE, which serve as key concepts that improve the understanding of the discipline within the research community [26]. The FHN model has been adopted to describe the essential dynamics of neuronal excitability but some aspects of the disturbed dynamics have not been studied extensively in experimentally realizable electrical circuits. In particular, the mathematical description of neuronal excitability and the reproduction of basic firing patterns have been the focus of most previous studies, while the explicit correlations between the mathematical state variables, physical circuit components, and experimentally observed neuronal-like dynamics are not necessarily well understood [27]. Furthermore, the ways in which external perturbations and parameter changes bring about transitions between different dynamical regimes (e.g., periodic, irregular, and chaotic-like behavior) must be explored systematically in more detail. A complete classification of the dynamical regimes in a parameter space with many dimensions is lacking, and it is challenging to correlate the theoretically predicted parameter regions with dynamically different types of neural-like dynamics and relate them to physical realizations of circuits.

To bridge these gaps, this paper proposes an experimentally feasible FHN-type neuron circuit using a two-capacitor configuration which includes piezoelectric components. The equations governing the dynamics are obtained from Kirchhoff’s laws of the electrical circuit and a direct link is provided between the variables of the circuit and the neuronal states. The proposed model is then systematically explored along a multi-dimensional parameter space to classify and map out the different dynamical regimes. This parameter space classification is applied to find regions of the parameter space which represent different behaviors, such as periodic, irregular, and chaotic-like dynamics, and to make a study of what effect is exerted by the external perturbations on the transitions between these different behaviors. Complementary tools used to characterize the identified dynamical states are time series analysis, phase portraits, return maps, power spectra, and statistical measures. Moreover, the proposed circuit is implemented in practice, and the signals measured experimentally are compared with their numerical simulation to check the experimental model and its modelization in the proposed circuit.

The results of this work are thus of four kinds. First, an FHN-type electrical neuron is developed using a two-capacitor and piezoelectric configuration and the mathematical model of the developed electrical neuron is established. Second, a systematic multidimensional parameter space classification is conducted to find and chart different dynamical regimes and their transitions. Third, the effect of external perturbations on these dynamical transitions is analyzed with the help of complementary nonlinear-dynamical methods such as time-series analysis, phase portraits, return maps, power spectra, and statistical measures. Fourth, the numerical prediction is compared to the experimental measurements carried out on the physical circuit, thus verifying the predicted dynamical behavior. By combining mathematical modeling, systematic categorization of the parameter space, dynamical analysis and experimental validation, this study offers a practical framework for investigating perturbed neuronal dynamics, and for exploring FHN-based circuits for neuromorphic applications.

The paper is organized as follows. The Mathematical Modelling and Experimental Validation section describes the mathematical model and its experimental validation. The categorization of the parameter space based on statistical properties and eigenvalue analysis is then presented. Finally, the dynamical behavior of the perturbed system is investigated.

### Mathematical modelling and experimental validation

The FHN circuit, an excitable non-linear electrical circuit, is widely used in non-linear dynamics and neuroscience studies [28]. Essentially, the circuit consists of an inductor, capacitors, a piezoelectric bi-membrane, resistors, and a non-linear resistor. Kirchhoff’s law is used to develop the governing equations, which characterize the dynamics of the circuit voltage and current components. The electrical diagram of the FHN circuit with a piezoelectric bimembrane is shown in Fig 1.


$$\begin{array}{c} \left\{ \begin{array}{cc} C_{1}\frac{dV_{1}}{dt} & =-\frac{V_{1}+V_{S}-V_{2}}{R_{S}},\\ C_{2}\frac{dV_{2}}{dt} & =\frac{V_{1}+V_{S}-V_{2}}{R_{S}}-I_{L}+\frac{1}{r}\left(V_{2}-\frac{1}{3}\frac{V_{2}^{3}}{V_{0}^{2}}\right),\\ L\frac{dI_{L}}{dt} & =-RI_{L}+E+V_{2}, \end{array}\right. \end{array}$$ (1)

where


$$\begin{array}{c} R_{S}=\left\{ \begin{array}{cc} r_{S}\left[1+\delta (V_{2}-V_{1})\right], & |V_{2}-V_{1}|\leq \tilde{\lambda },\\ r_{S}(1+\delta \tilde{\lambda }), & |V_{2}-V_{1}|>\tilde{\lambda }. \end{array}\right. \end{array}$$

**Fig 1:**
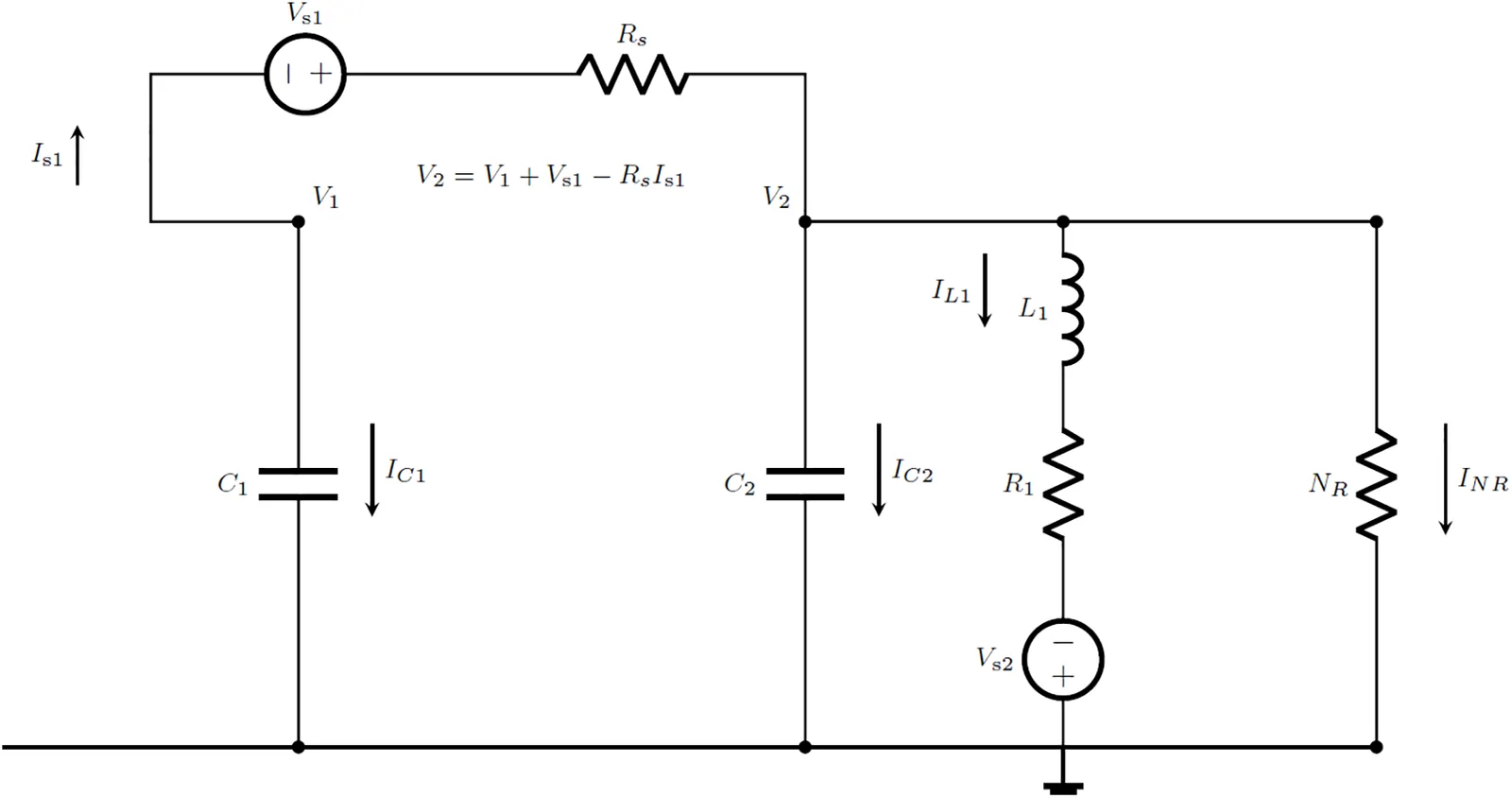
The electrical diagram of the FHN circuit with a piezoelectric bimembrane.

The capacitor voltages *V*_1_ and *V*_2_ represent the electrical membrane potentials associated with different neuronal membrane states. The capacitances *C*_1_ and *C*_2_ represent effective membrane capacitances responsible for storing electrical charge across the corresponding membrane regions. The inductor current $I_{L}$ represents an internal dynamical current associated with neuronal ionic or recovery dynamics. The resistance *R* represents an effective dissipative or leakage pathway, while the external voltage *E* represents an externally applied electrical stimulus. The source voltage $V_{S}$ represents an external or coupling input to the neuronal system. Thus, the circuit variables provide an electrical analogue of the principal voltage, current, recovery, and external-stimulation processes involved in neuronal dynamics. The circuit is made up of two capacitors, *C*_1_ and *C*_2_, which dynamically store and release charge. A non-linear resistor $R_{S}$ introduces nonlinearity by varying its resistance in response to voltage variations. The time dependence is introduced into the current via an inductor *L*, which stores energy in its magnetic field. The behavior of the circuit is affected by a piezoelectric voltage source *V*, which functions as a function of time. Provide a reference potential and load using a parallel voltage source *E* and resistance *R*. In Eq. (1), $r_{s}$ represents the base resistance. δ represents the modulation factor for small voltage differences. λ denotes the threshold voltage difference. $i_{\lambda }$ represents an imaginary term for high voltage differences.

The circuit may be modeling excitable systems, like neurons, or it may be a component of an oscillator with non-linear feedback dynamics, as suggested by the interaction of the capacitive, inductive, and resistive components. The circuit can simulate electrical pulses or wave propagation in neural networks because the inductor and resistors control energy flow, while the capacitors add time-dependent behavior. Node analysis is essential to understanding the interactions between various components in the voltage and current flow analysis of this circuit. The voltage differential across $R_{S}$ determines the current flowing through the capacitor *C*_1_ at node *V*_1_, which affects the charge flow. The current flows through the capacitor *C*_2_ and inductor *L* at node *V*_2_, where the voltage controls $I_{L}$ using an inductive differential equation. With $I_{L}$ flowing towards the resistor *R*, the current moves from *V*_1_ to *V*_2_ through $R_{S}$. By dynamically influencing *V*_1_ and *V*_2_, the piezoelectric voltage source $V_{S}$ modifies the voltage distribution and current flow, influencing both the transient and steady-state behavior of the system.

The following dimensionless transformation is introduced to simplify and analyze the system [29]:


$$\begin{array}{c} \left\{ \begin{array}{cc} p_{1,2} & =\frac{V_{1,2}}{V_{0}},\;T=\frac{t}{rC_{2}},\;u_{s}=\frac{V_{S}}{V_{0}},\;q=\frac{C_{1}}{C_{2}},\;\delta =\tilde{\delta }V_{0},\;\lambda =\frac{\tilde{\lambda }}{V_{0}},\\ G & =\frac{r}{R_{S}}=\left\{ \begin{array}{cc} \frac{r}{r_{S}\left[1+\delta (x_{2}-x_{1})\right]}, & |x_{2}-x_{1}|\leq \lambda ,\\ \frac{r}{r_{S}(1+\delta \lambda )}, & |x_{2}-x_{1}|>\lambda , \end{array}\right.\\ v & =\frac{r}{V_{0}}I_{L},\;k=\frac{E}{V_{0}},\;l=\frac{R}{r},\;m=\frac{C_{2}r^{2}}{L}. \end{array}\right. \end{array}$$ (2)

Using these, the differential equation becomes:


$$\begin{array}{c} \left\{ \begin{array}{cc} \frac{dp_{1}}{dT} & =-\frac{G}{q}(p_{1}-p_{2}),\\ \frac{dp_{2}}{dT} & =G(p_{1}+u_{S})-v+(1-G)p_{2}-\frac{1}{3}p_{2}^{3},\\ \frac{dv}{dT} & =m(k+p_{2}-lv), \end{array}\right. \end{array}$$ (3)

The parameters introduced in Eq. (3) are dimensionless quantities obtained through the nondimensionalization procedure. Specifically, $q=C_{1}/C_{2}$ represents the capacitance ratio, $G=r/R_{S}$ denotes the normalized conductance, *k* = *E*/*V*_0_ represents the normalized external voltage parameter, *l* = *R*/*r* is the normalized resistance parameter, and $m=C_{2}r^{2}/L$ corresponds to the normalized inductive parameter. Therefore, a*ll* parameters are dimensionless and characterize the relative contribution of different circuit components to the system dynamics.

In this work, *G*, *q*, *m*, *k*, and *l* are considered as control parameters for investigating the nonlinear dynamical behavior of the system. Although $q=C_{1}/C_{2}$ corresponds to a positive capacitance ratio in a conventional passive circuit, negative values of *q* are considered here within a generalized mathematical framework to explore the influence of effective capacitance effects on the system dynamics. Similarly, negative values of *G* represent effective negative conductance conditions, which can be realized using active circuit configurations such as negative impedance converters. The parameter ranges adopted in the numerical simulations are G∈[range], q∈[range], m∈[range], k∈[range], and l∈[range]. The chosen dynamical behavior of the system is provided by the parameter selections. In the system of Eq. (3), the non-autonomous system simplifies to the autonomous version shown below when $u_{S}=0$:


$$\begin{array}{c} \left\{ \begin{array}{cc} \frac{dp_{1}}{dT} & =-\frac{G}{q}(p_{1}-p_{2}),\\ \frac{dp_{2}}{dT} & =Gp_{1}-v+(1-G)p_{2}-\frac{1}{3}p_{2}^{3},\\ \frac{dv}{dT} & =m(k+p_{2}-lv). \end{array}\right. \end{array}$$ (4)

A perturbation term, where *Q* is the amplitude and *w* is the angular frequency of the perturbed term, will be added to the Eq. (4) in order to examine different patterns. The perturbation term evaluates the robustness and sensitivity of the system by simulating realistic disturbances. The Eq. (4) with the perturbed term looks like this:


$$\begin{array}{c} \left\{ \begin{array}{cc} \frac{dp_{1}}{dT} & =-\frac{G}{q}(p_{1}-p_{2}),\\ \frac{dp_{2}}{dT} & =Gp_{1}-v+(1-G)p_{2}-\frac{1}{3}p_{2}^{3},\\ \frac{dv}{dT} & =m(k+p_{2}-lv)+Q\cos(n),\\ \frac{dn}{dT} & =w. \end{array}\right. \end{array}$$ (5)

The proposed system is derived from an equivalent nonlinear piezoelectric bi-membrane circuit, where the variable *v* represents the normalized inductor current associated with the electromechanical energy transfer pathway. In a piezoelectric structure, external mechanical excitation (such as pressure or acoustic loading) generates an induced electric response through the direct piezoelectric effect. This electromechanical coupling is represented as an external current/flux excitation rather than a direct modification of the capacitive voltage variables. Therefore, the periodic forcing term Qcos(n) is introduced into the *v*-equation, which describes the current evolution and energy exchange dynamics of the equivalent circuit. The voltage variables *p*_1_ and *p*_2_ describe the internal electrical states of the nonlinear circuit, while the variable *v* captures the dynamic response associated with the energy-storage element and external excitation pathway. Introducing the forcing term in the *v*-equation allows the model to represent an externally driven piezoelectric response while preserving the intrinsic nonlinear voltage dynamics of the system. The goal is to comprehend how minor pressure like modifications impact the dynamics of the model, providing insight on neuron stability. Although a number of analytical methods, such as power spectrum and return maps, are discussed, distinct figures are required to show behaviors across parameter regions. Using a variety of techniques, we examine the behavior of a dynamical system in this work. To simulate the real output of a biological neuron, we use dynamical models made up of a non-linear set of differential equations to describe neuronal response and behavior [30,31].

The measured signals were subsequently processed to obtain the corresponding phase plots presented in Fig 2. Figure presents the experimentally obtained Nyquist plots [32] under different excitation voltages and frequencies. The observed trajectories exhibit qualitative agreement with the theoretical and Multisim results, indicating that the implemented circuit successfully reproduces the expected nonlinear dynamical behavior over the investigated operating conditions. These experiments demonstrate the practical feasibility of the proposed circuit and provide qualitative experimental support for the theoretical analysis. The Nyquist plots represent the magnitude and angle of the capacitor voltages $V_{C_{1}}$ and $V_{C_{2}}$, for various frequencies and driving amplitudes of the excitation. Each subplot shows $V_{C_{2}}$ as a function of $V_{C_{1}}$, allowing us to gain insight into the dynamic response of the circuit in each subplot. The frequencies of excitation were chosen as 800 *Hz*, 1000 *Hz*, 1500 *Hz*, 1700 *Hz* and peak to peak driving voltage was selected as 8*V*, 16*V*. The values of the above parameters were chosen to explore the effect of frequency and amplitude of excitation on the dynamics of the system to see if different regimes of operation could be discovered. Fig 2a and 2b (with *f* = 800 Hz) show closed, relatively regular orbits, suggestive of stable oscillations. The orbits become increasingly distorted and cover a larger area on the phase plane as the frequency of the exciting force is increased to 1000 Hz, 1500 Hz, and 1700 Hz (Fig 2c-2h). In this behavior, more complex nonlinear dynamics are observed, as well as increased interaction between the circuit components. Comparing the results for $V_{pp}=8$V and $V_{pp}=16$V, one can see that the geometry of the trajectories changes with increasing driving amplitude and the nonlinear response of the circuit is improved. The larger and more complex loops at higher amplitudes suggest increased energy transfer between the components of the system and increased sensitivity to the excitation conditions. Both the amplitude of the driving signal and its frequency are found to show significant influence on the dynamics of the circuit, and can induce transitions between regular and more complicated oscillatory regimes. In general, the Nyquist plots demonstrate that the circuit is indeed non-linear and validate the experimental results of dramatic differences in system behavior when the control variables are altered.

**Fig 2:**
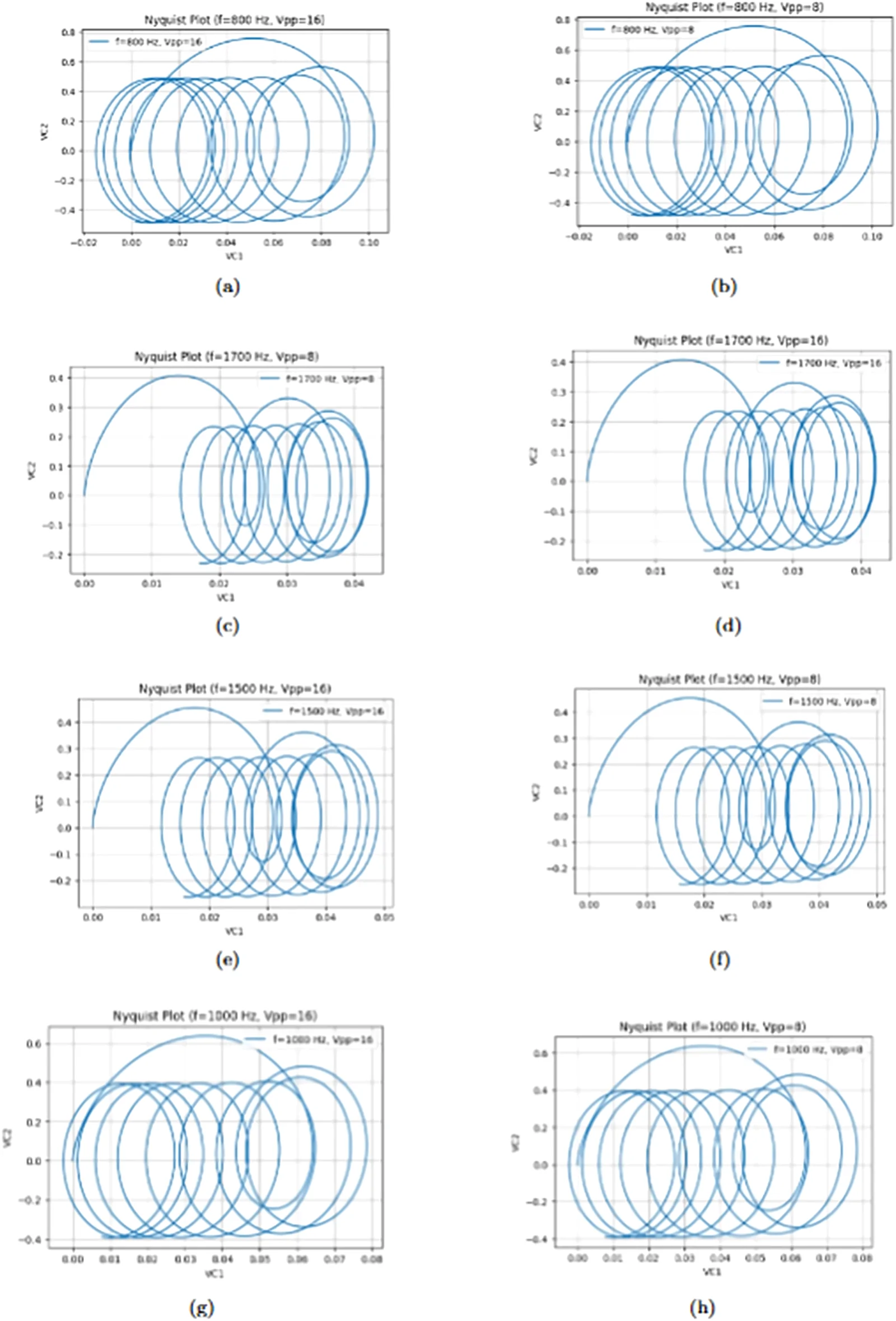
Nyquist plots of the circuit response for different excitation frequencies and applied voltage amplitudes.

The Multisim implementation of the proposed FHN circuit is displayed in Fig 3. The circuit consists of linear and nonlinear components, including operational amplifiers, resistors, capacitors, and diodes, which are used to reproduce the fundamental dynamics of the FHN neuron model. The capacitive elements are the state variables of the system, and the resistive and nonlinear elements are the mechanisms of excitation and recovery that lead to neuron-like behavior. The goal of the design for the circuit was to implement the FHN equations electronically and to allow investigation of the excitable dynamics numerically. The circuit can be made to produce voltage responses similar to biological action potentials by choosing suitable values of the components and the inputs. The nonlinear feedback paths give rise to the necessary thresholds and recovery properties and allow for the system to have many different types of dynamics. The Multisim model is used to test the predicted theoretical results according to the FHN system and investigate the role of the circuit parameters on the overall dynamics of the system. The change in both the amplitude and frequency of the oscillations, along with the change in the structure of the oscillations (waveform), can vary significantly with the change in the values of the components and the voltage of the exciting source. Hence, the circuit can be used as an effective tool for studying nonlinear phenomena in excitable systems and for investigating application research in neuromorphic engineering, artificial neural networks and brain-inspired computing architectures.

**Fig 3:**
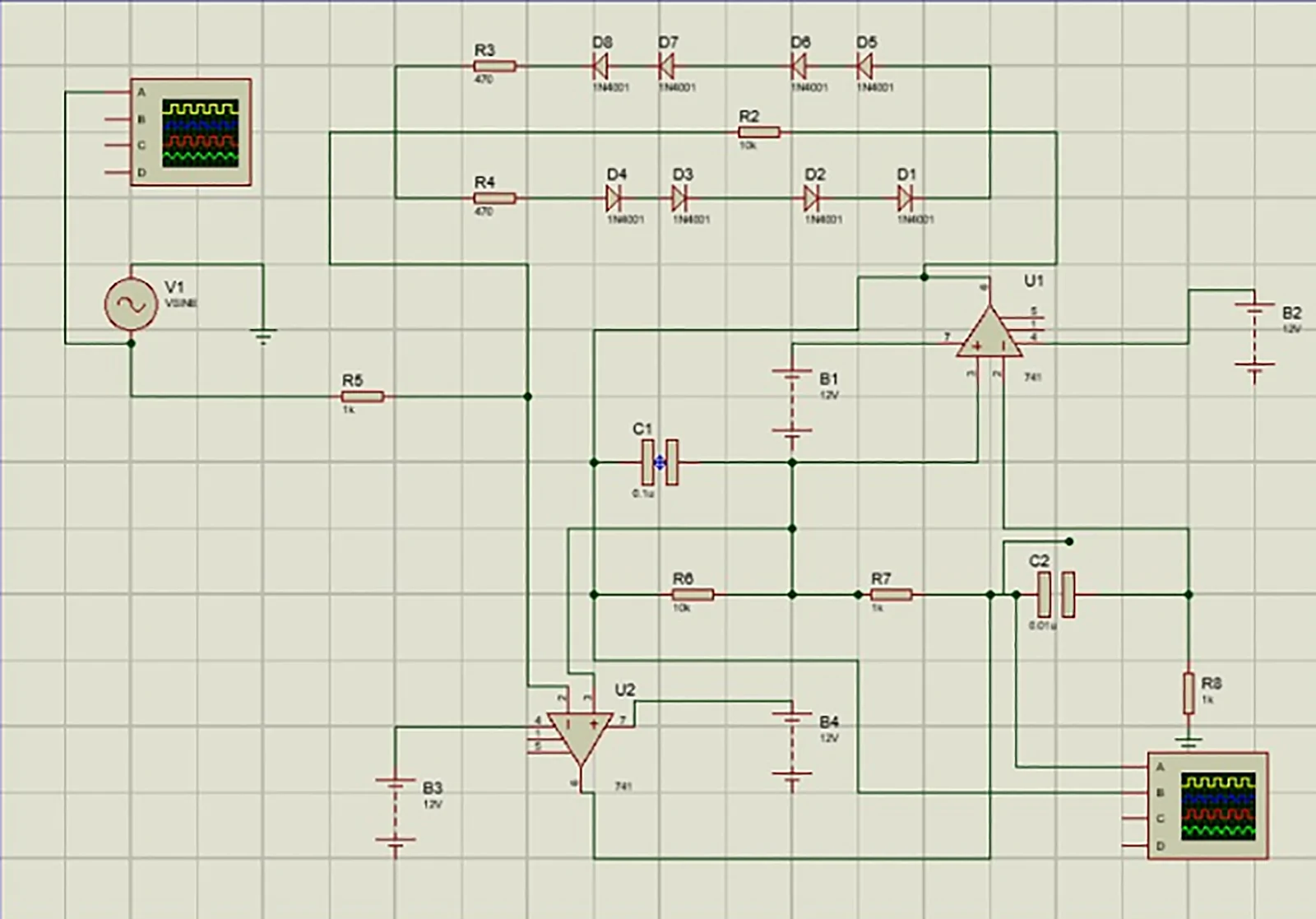
Multisim implementation.

### Energy-function analysis

To further characterize the energetic behavior of the proposed neuron circuit, an energy-based analysis is introduced following the Hamilton energy formulation discussed by Yang et al. [33]. In realistic nonlinear neural circuits, the energy associated with capacitive and inductive elements can be used to construct a Hamilton energy function, which provides a physical interpretation of the energy exchange and dissipation processes in the circuit. The energy formulation is particularly useful for distinguishing energy storage, dissipation, and external energy injection in nonlinear neuron circuits [34].

For the dimensional circuit model in Eq. (1), the Hamilton energy is constructed from the two capacitive variables and the inductive variable as:


$$\begin{array}{c} W_{H}=-\frac{1}{2}C_{1}V_{1}^{2}+\frac{1}{2}C_{2}V_{2}^{2}+\frac{1}{2}LI_{L}^{2}. \end{array}$$ (6)

The signed contribution of the first capacitive variable follows the Hamilton-energy representation used for active nonlinear neural circuits and should not be interpreted as negative physical stored electromagnetic energy. Rather, it is part of the signed Hamilton energy associated with the dynamical representation of the active circuit [35].

Using the dimensionless variables and parameters and normalizing Eq (6) by $C_{2}V_{0}^{2}$, the dimensionless Hamilton energy function becomes:


$$\begin{array}{c} H(p_{1},p_{2},v)=-\frac{q}{2}p_{1}^{2}+\frac{1}{2}p_{2}^{2}+\frac{1}{2m}v^{2}. \end{array}$$ (7)

the energy associated with the neuron is determined by the capacitive states *p*_1_ and *p*_2_ and the inductive state *v*. The additional phase variable *n* introduced for periodic forcing does not constitute an independent energy-storage element and therefore does not appear explicitly in Eq. (7).

For the unforced system in Eq. (4), differentiation of Eq. (7) with respect to the dimensionless time *T* gives:


$$\begin{array}{c} \frac{dH}{dT}=-qp_{1}\frac{dp_{1}}{dT}+p_{2}\frac{dp_{2}}{dT}+\frac{v}{m}\frac{dv}{dT}. \end{array}$$ (8)

Substitution of Eq. (4) into Eq. (8) yields:


$$\begin{array}{c} \frac{dH}{dT}=Gp_{1}^{2}+(1-G)p_{2}^{2}-\frac{1}{3}p_{2}^{4}+kv-lv^{2}. \end{array}$$ (9)

Eq. (9) represents the instantaneous energy-balance rate of the unforced neuron. The terms *kv* and $-lv^{2}$ respectively describe energy supplied by the voltage source and energy dissipated through the linear resistance. The nonlinear contribution $\left(p_{2}^{2}-p_{2}^{4}/3\right)$ represents state-dependent energy generation and dissipation associated with the nonlinear FHN-type element. Consequently, the energy rate is not required to remain negative, reflecting the active nature of the neuron circuit.

For the periodically forced model in Eq. (5), the voltage-like variable satisfies:


$$\begin{array}{c} \frac{dv}{dT}=m\left(k+p_{2}-lv\right)+Q\cos(n),\;\frac{dn}{dT}=w. \end{array}$$ (10)

Consequently, the corresponding energy-balance rate becomes:


$$\begin{array}{c} \frac{dH}{dT}=Gp_{1}^{2}+(1-G)p_{2}^{2}-\frac{1}{3}p_{2}^{4}+kv-lv^{2}+\frac{Q}{m}v\cos(n). \end{array}$$ (11)

The final term,


$$\begin{array}{c} P_{ext}=\frac{Q}{m}v\cos(n), \end{array}$$ (12)

is the instantaneous contribution of the external periodic forcing to the energy balance. Since $\frac{dn}{dT}=w$, the forcing phase can be expressed as:


$$\begin{array}{c} n=n_{0}+wT, \end{array}$$ (13)

giving:


$$\begin{array}{c} \frac{dH}{dT}=Gp_{1}^{2}+(1-G)p_{2}^{2}-\frac{1}{3}p_{2}^{4}+kv-lv^{2}+\frac{Q}{m}v\cos\left(n_{0}+wT\right). \end{array}$$ (14)

Therefore, the energy analysis provides a complementary physical interpretation of the dynamical response. Positive values of *dH*/*dT* correspond to instantaneous net energy injection into the circuit, whereas negative values indicate instantaneous net energy dissipation. The alternating positive and negative energy-balance rates observed in Fig 4 continuous energy exchange among the nonlinear circuit components and the external forcing.

**Fig 4:**
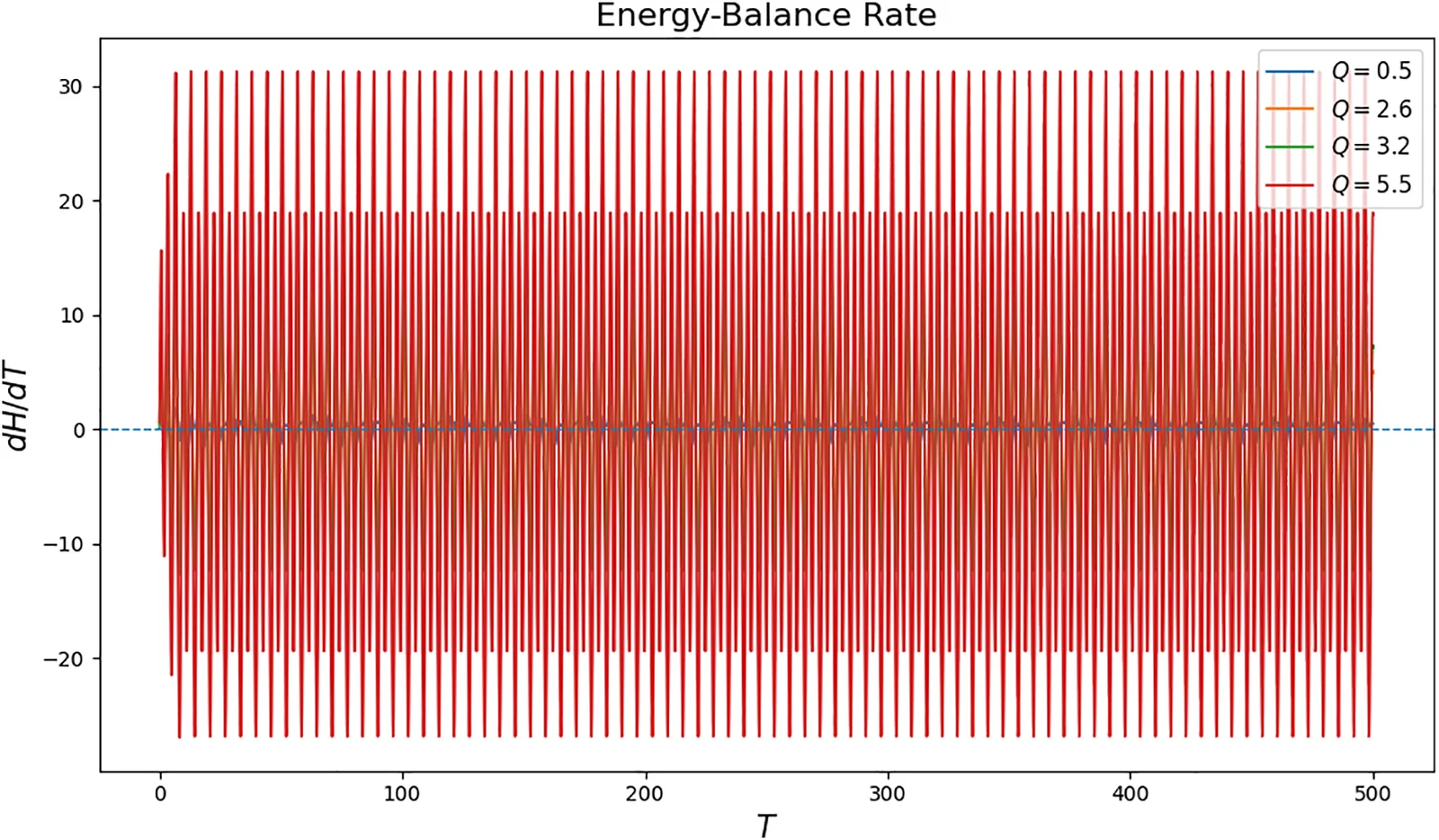
Energy-balance rate $\frac{dH}{dT}$ of the forced neuron circuit.

Increasing the forcing amplitude *Q* enhances the forcing-dependent contribution Qvcos(n)/m, thereby modifying the instantaneous energy exchange and the resulting dynamical response. It should be emphasized that the Hamilton energy in Eq. (7) is used here as an energetic diagnostic and is not claimed to be a positive-definite Lyapunov function.

### Systematic parameter space categorization

This research aims to monitor and control modifications in complex dynamic processes. The research adopts parameter space categorization as its methodology to create different combinations to examine system responses. We conducted a systematic parameter space categorization based on three structured steps for examining dynamical systems. First, we established a search space. Then, we determined parameter classes through statistical system response properties, including maximum or minimum values, to distinguish between diverging, non-diverging, and periodic patterns. This step detects unstable zones while mapping stable oscillatory patterns for different starting points. We identify stability regions through the analysis of the eigenvalues of the Jacobian matrix by interpreting a zero or positive eigenvalue *R*4 as marginal stability. In contrast, complex eigenvalues with negative real parts signify *RC*2 as damped oscillatory behavior.

### Search space

This research uses a systematic framework to evaluate the dynamical system for extensive parameter variations. First, we determine the acceptable values for each parameter to implement the systematic framework for the dynamical system described in Eq. (5). The system incorporates three main parameters, including *G*, *q*, *m* ranging from −2 to 2 and receives discrete values 10. In addition, the fixed parameters are set as (*k*, *l*, *w*, *Q*) = (0.3, 1, 3, 2). Although, these dimension*l*ess parameters are defined by physical circuit quantities and physically expected to be positive; however, the use of negative values is only for theoretical and mathematical exploration of the parameter space, and should not be interpreted as physically realizable operating conditions of the electronic circuit. The choice of the search interval [−2,2] was made to allow for a fairly wide range of exploration of the global dynamics of the governing nonlinear model in a systematic manner. This range allows for recognition of the transitions between the various dynamical behaviors and can help attain a complete characterization of the parameter space, as was done in previous work with nonlinear oscillatory systems [36]. The three-dimensional space of parameter values becomes the center of interest because every single point represents a different combination. The model contains a thousand possible combinations for each starting condition that needs testing. The total search space originates from a combination of parameter sets amounting to |G×q×m| and creates potential cases 1000 due to the given parameter range defined for the dynamical system. Note that identical combinations are made for three initial conditions $\left(p_{1},p_{2},v,n\right)$ = (2, 0.5, 1, 2), (2, 4, 1, 3), and (1, 2, 0.3, 0.2). The search space for the parameters *G*, *q*, and *m* is shown in Fig 5.

**Fig 5:**
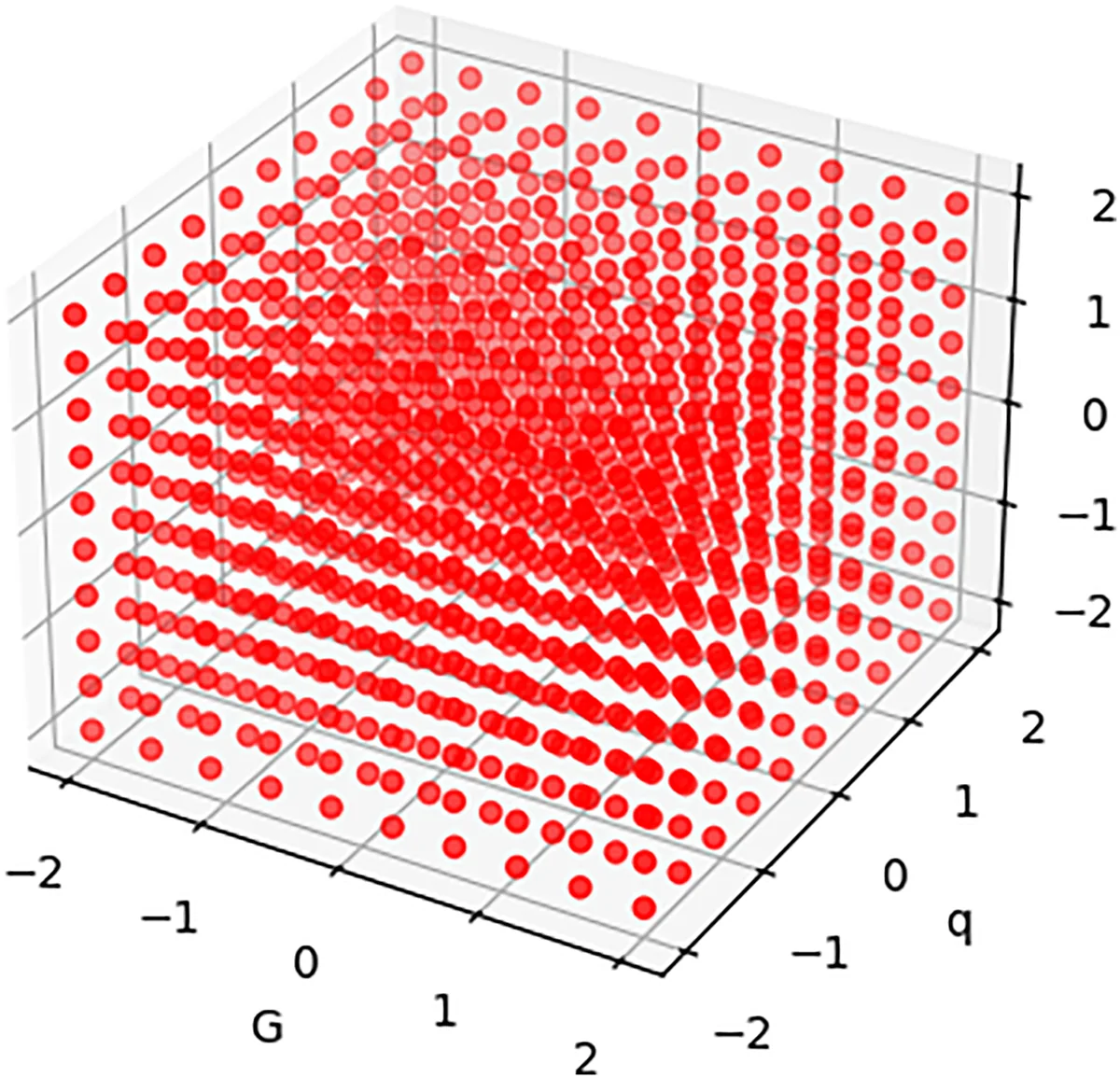
Search space by taking parameters *G* = (−2,2), *q* = (−2,2), *m* = (−2,2).

### Categorization based on statistical properties

The analysis describes the classification of the parameter space of the dynamical system into different zones based on the defined statistics, such as the maximum and minimum values, by setting a threshold at $1\times 10^{6}$ for divergent cases and $1\times 10^{-3}$ for non-divergent cases. The chosen thresholds were chosen to be simple and easy to compute numerically to explore the parameter space. In numerical simulations, infinite values were not allowed to represent divergence, so a threshold of $1\times 10^{6}$ was used as a cutoff point to determine if the trajectory was unbounded or if the response was bounded. On the other hand, an absolute tolerance of $1\times 10^{-2}$ was used to find the steady state solutions by setting the absolute difference between the maximum and minimum values of each state variable to be less than this. The remaining bounded trajectories were classified as periodic based on the recurrence of successive peaks, indicating repeating oscillatory behavior. The parameters were varied within the specified range for *G*, *q*, and *m*, and the behavior of the resulting system was classified into three types: divergent, non-divergent, and periodic. This particular categorization helps to capture the behavior of the system under different conditions. The response of the system was tested with three different initial conditions: $\left(p_{1},p_{2},v,n\right)$ = (2, 0.5, 1, 2), (2, 4, 1, 3), and (1, 2, 0.3, 0.2). As shown in Fig 6, the system exhibits divergent behavior, with a small fraction displaying periodic behavior. The results indicate that the system demonstrates unstable states, as divergent behavior emerges as the principal outcome from all initial conditions and parameter values tested. Only periodic oscillations occurred in some cases among all initial conditions, yet the system demonstrably avoided stable non-diverging states. Table 1 describes the categories of classification of the dynamical behavior of the system, and Table 2 highlights the categorization for various initial conditions. The system displays periodic oscillations and divergent behavior, which are periodic only for small values. This system is more sensitive to parametric disturbances than to changes in initial values. The sensitivity of the system leads to a similar response across various initial conditions, and the primary outcome is instability. Changes in parameters cause the system to enter an unstable state and exhibit oscillations rather than steering it toward stable equilibrium states. It has been found that the system exhibits strong sensitivity to changes in system parameters; therefore, it is essential to note the effects of these parameters on the system dynamics, especially in non-linear systems. The analysis provides a basis for further studies on stabilization techniques for these systems, including starting setups and how the parameters control the system behavior. Additional tests indicate that more predictable and stable results can be achieved through the systematic adjustment of control parameters.

**Table 1:** Category and description of statistical properties.

Category	Description
**Total**	Total number of cases analyzed
**Diverging**	$|p_{1_{\text{max}}}|,|p_{1_{\text{min}}}|,|p_{2_{\text{max}}}|,|p_{2_{\text{min}}}|,|v_{\text{max}}|,|v_{\text{min}}|,|n_{\text{max}}|,|n_{\text{min}}|>\text{threshold}$
**Non-diverging**	$|p_{1_{\text{max}}}-p_{1_{\text{min}}}|,|p_{2_{\text{max}}}-p_{2_{\text{min}}}|,|v_{\text{max}}-v_{\text{min}}|,|n_{\text{max}}-n_{\text{min}}|<\text{threshold}$
**Periodic**	Repeating oscillatory behavior identified from successive peaks

**Table 2:** Categorization for various initial conditions.

Category	Initial condition (2, 0.5, 1, 2)	Initial condition (2, 4, 1, 3)	Initial condition (1, 2, 0.3, 0.2)
**Total**	1000	1000	1000
**Diverging**	651	648	649
**Non-diverging**	0	0	0
**Periodic**	349	352	351

**Fig 6:**
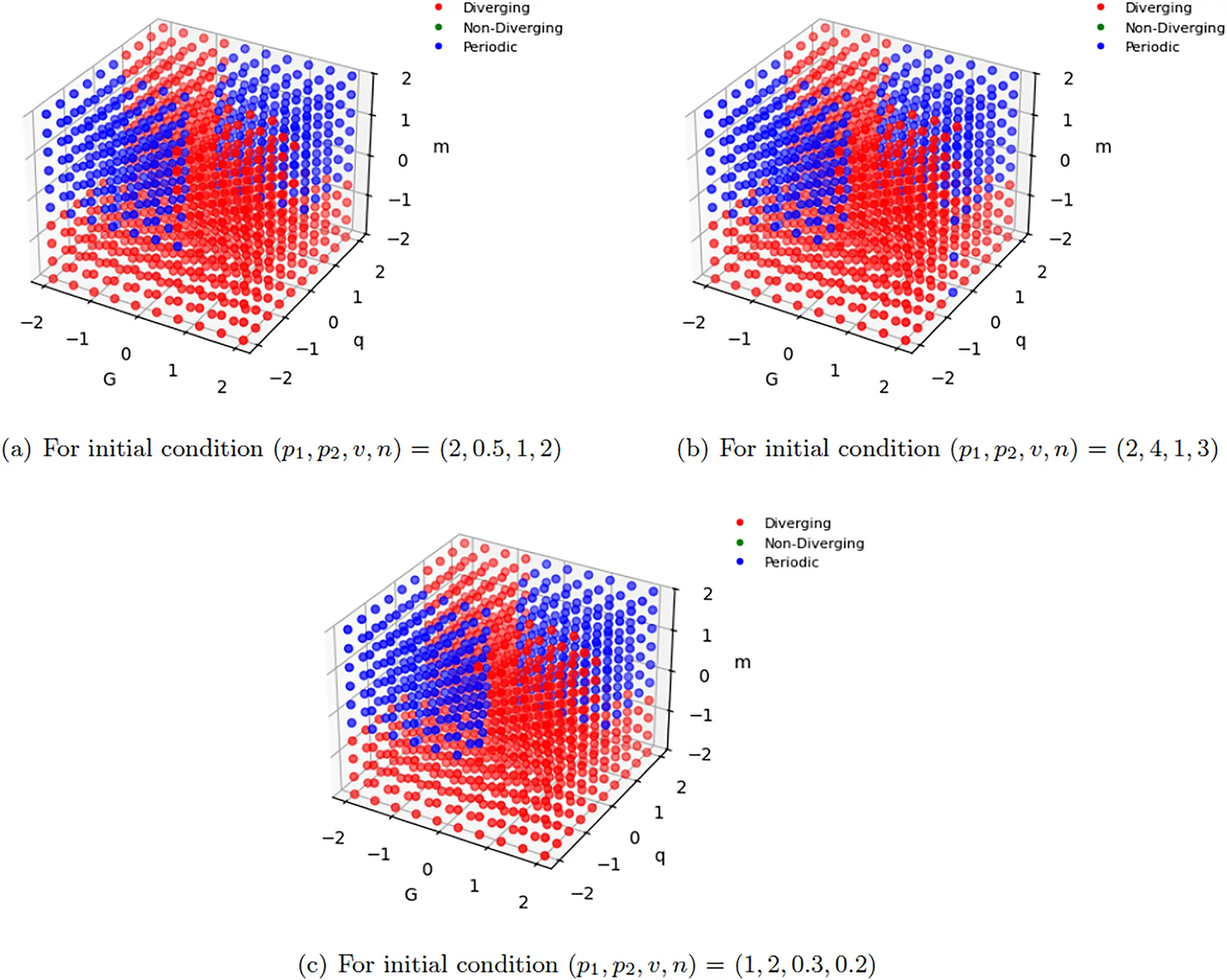
Categorization based on statistical properties by taking different initial conditions for parameters G∈(−2,2), q∈(−2,2), m∈(−2,2), and (*k*, *l*, *w*, *Q*) = (0.3,1,3,2).

### Categorization based on eigenvalues

The behavior of dynamical systems is studied using advanced eigenvalue techniques to identify the numerous stability regimes and dynamical features that appear in different parts of the parameter space. By doing this, it is possible to gain more insight into the nature of systems with respect to the way they can respond to different conditions that characterize the motion of stability and dynamic transformation. This analysis will ensure that the dynamic behavior of the system has been thoroughly covered; we have selected different parameter values as well as initial states to understand the stability of the system across a broad range of configurations. Specifically, the parameters were scanned in the ranges of G, q, and m, which belong to the interval of −2 to 2, respectively. These values were selected to cover the low and high dynamical regimes of the system. In the eigenvalue-based categorization, the Jacobian matrix was evaluated at the selected initial condition corresponding to each simulation rather than at a single fixed equilibrium point. Three representative initial conditions, namely (2, 0.5, 1, 2), (2, 4, 1, 3), and (1, 2, 0.3, 0.2), were considered to investigate how the local linear characteristics vary with different initial states. This makes it possible to systematically investigate the local linear stability of the system as the parameters and initial conditions are varied. The Jacobian matrix was computed for each parameter combination (*G*,*q*,*m*) with the corresponding initial condition, and the eigenvalues of each Jacobian matrix were calculated to determine the local linear behavior. First, the eigenvalues are calculated using Eq. (5), and then the different classes of eigenvalues are shown in Table 3, while the summary of the eigenvalue categories with the corresponding initial conditions is shown in Table 4. In Fig 7 the categories of eigenvalues achieved for various parameter sets and for each of the three initial conditions are displayed. Parameter space maps are provided for each to show how the local linear properties change with initial state. The 3*D* plots demonstrate that each point represents a unique adjustment between the parameters *G*, *q*, and *m*. This system displays each point with different colors to show the eigenvalue categories found in the selected parameter settings. *B*1 represents the region where all eigenvalues remain greater than zero, while *B*2 indicates where all eigenvalues fall between zero and negative integers. Furthermore, the area that contains complex eigenvalues with negative real parts is labeled *BD*2. The categories *B*2 and *BD*2 contain stable systems, while unstable systems are found in *B*1, *B*3, and *BD*1. Periodic behavior occurs in categories *D*, *B*4, and *B*5 with oscillations or marginal stability. The critical aspect of this method is that these graphs have been generated using different initial conditions for the underlying system. The initial conditions were changed to examine the local linear behaviour of the system for various regions of the Jacobian matrix in the state space. The observed variations show that different initial conditions affect the boundaries of regions corresponding to different eigenvalue categories, revealing the complex relationship between parameter choice, initial states, and system dynamics.

**Table 3:** Summary of eigenvalue categories and their nature.

Tag	Eigenvalues	$\lambda_{1}$, $\lambda_{2}$ nature
***B*1**	All eigenvalues > 0	$\lambda_{1}\lambda_{2}=a,a$
***B*2**	All eigenvalues < 0	$\lambda_{1}\lambda_{2}=-a,-a$
***B*3**	Mixed sign real eigenvalues	$\lambda_{1}\lambda_{2}=\pm a$
***B*4**	One zero, one positive	$\lambda_{1}\lambda_{2}=0,a$
***B*5**	One zero, one negative	$\lambda_{1}\lambda_{2}=0,-a$
***D***	Purely imaginary	$\lambda_{1}\lambda_{2}=\pm bi$
***BD*1**	Complex with real > 0	$\lambda_{1}\lambda_{2}=a\pm bi$
***BD*2**	Complex with real < 0	$\lambda_{1}\lambda_{2}=-a\pm bi$

**Table 4:** Summary of eigenvalue categories with corresponding initial conditions.

Tag	Initial condition (2, 0.5, 1, 2)	Initial condition (2, 4, 1, 3)	Initial condition (1, 2, 0.3, 0.2)
***B*1**	0	0	0
***B*2**	0	0	0
***B*3**	0	0	0
***B*4**	476	734	513
***B*5**	17	232	75
***D***	0	0	0
***BD*1**	400	16	237
***BD*2**	107	18	175

**Fig 7:**
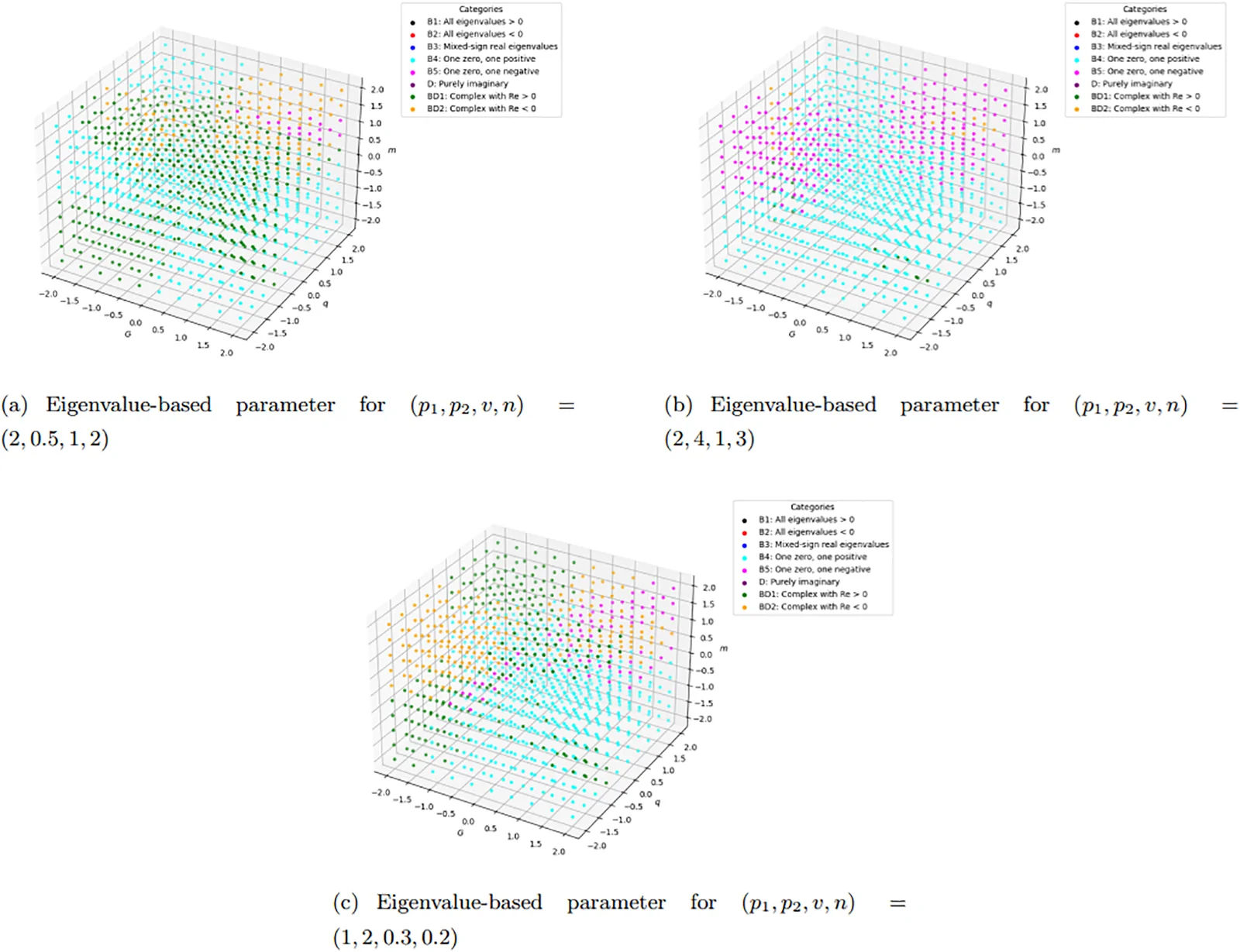
Categorization based on eigenvalues for various initial conditions. Parameters: G,q,m∈(−2,2), (*k*,*l*,*w*,*Q*) = (0.3,1,3,2).

### Ensemble-size sensitivity analysis

To evaluate the robustness of the proposed statistical characterization framework, an ensemble-size sensitivity analysis was performed. The goal was to see if the statistical properties used for categorization of a parameter space are statistically significant with the number of initial conditions sampled. For this purpose, ensembles consisting of *N* = 50, 100, 150, and 200 initial conditions were taken into account using nested ensembles. The number of trajectories was determined for each trajectory, i.e., $\log_{10}(r_{\max}+1)$, where $r_{\max}$ is the maximum radius achieved in the simulation. Empirical cumulative distribution functions (CDFs) and kernel density estimates (KDEs) were used to analyze the resulting distributions. The ensemble-size sensitivity analysis results for two typical parameter sets are shown in Fig 8. The left column plots the empirical CDFs and the right column plots the KDEs for various numbers of ensembles. As the number of ensemble members grows, the CDFs become more overlapped, which demonstrates convergence towards a stable cumulative distribution. Similarly, the KDE curves converge more and more as *N* increases, indicating that the estimated probability density functions are less influenced by sampling variability for larger *N*. The observed convergence behavior shows that the statistical descriptors used in the categorization framework are rather insensitive to the choice of ensemble size.

**Fig 8:**
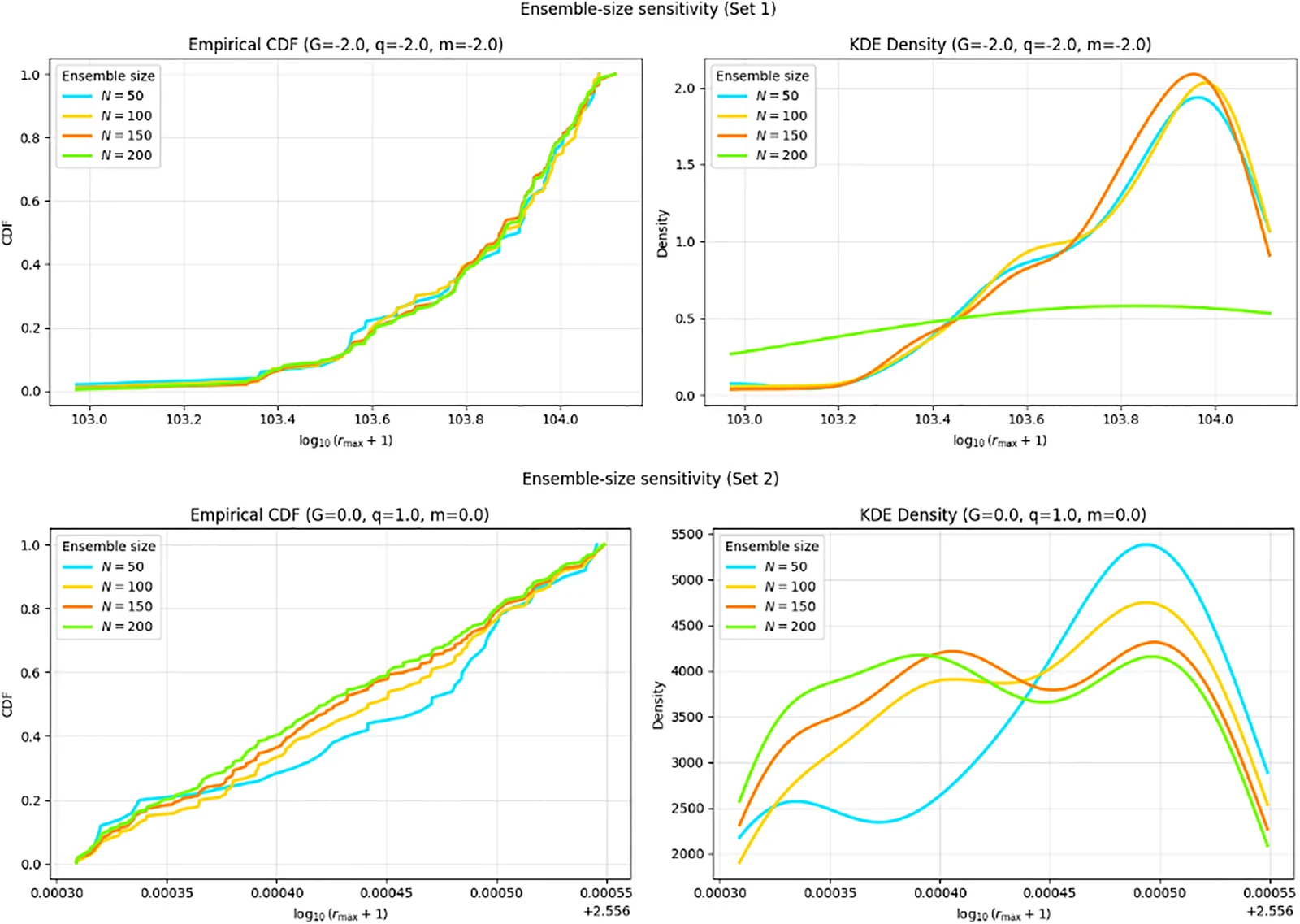
Ensemble-size sensitivity analysis for two representative parameter sets. The left column shows the empirical cumulative distribution functions (CDFs), while the right column presents kernel density estimates (KDEs) of $\log_{10}(r_{\max}+1)$ for different ensemble sizes *N* = 50,100,150, and 200.

The differences are only slightly noticeable for smaller ensembles but decrease as more initial conditions are added to the analysis. This trend reflects that the extracted statistical properties are representative of the intrinsic dynamics of the system and are not caused by the lack of sampling. Moreover, the ensemble-size sensitivity analysis also gives some idea of the sampling needs of the proposed approach. The more initial conditions that are sampled, the smoother and more stable the estimated distributions are, which means that this is statistically more reliable. A decrease in the fluctuations of the CDF and KDE curves as a function of the ensemble size indicates that the most important dynamical parameters of the system are reasonably well represented by reasonably large ensembles. Therefore, adding extra trajectories beyond a certain limit does not lead to significant changes in the estimated distributions. The results, therefore, validate the robustness and reproducibility of the statistical characterization procedure.

### Dynamical analysis

In this section, we will analyze the dynamics of the FHN model [37,38]. The stability evaluations of the models will be achieved by rapid estimations of the power spectrum [39], the return map, the noise detection map, and the disturbance profile for various initial conditions, which will be addressed in our standard examines. The system responses can be understood through analysis of the disturbance movements such as the stable and unstable and potentially chaotic motions. The results are amazing and different with regard to the viewing and revealing of underlying mechanisms involved.

### Power spectrum

The two initial conditions were used to create the power spectra shown in Fig 9. This figure shows that, in the one shown in Fig 9a, the high-frequency components have a similar decay rate, but the low-frequency components are different in the two trajectories. This suggests that the initial conditions primarily affect low frequencies and that the higher frequencies are relatively constant. The power of the spectra is focused at a few frequencies, which is characteristic of a predominantly periodic dynamics. The spectra in Fig 9b starts to get closer to each other and extra intermediate-frequency components can be seen on the red trajectory. These components represent nonlinear harmonic generation and interaction between characteristic frequencies. The nonlinear terms may redistribute the energy from the dominant frequency to higher harmonics, especially that of *q* and *G*. The resulting discrete spectral structure is consistent with a more complex oscillatory behavior, but not necessarily with chaos.

**Fig 9:**
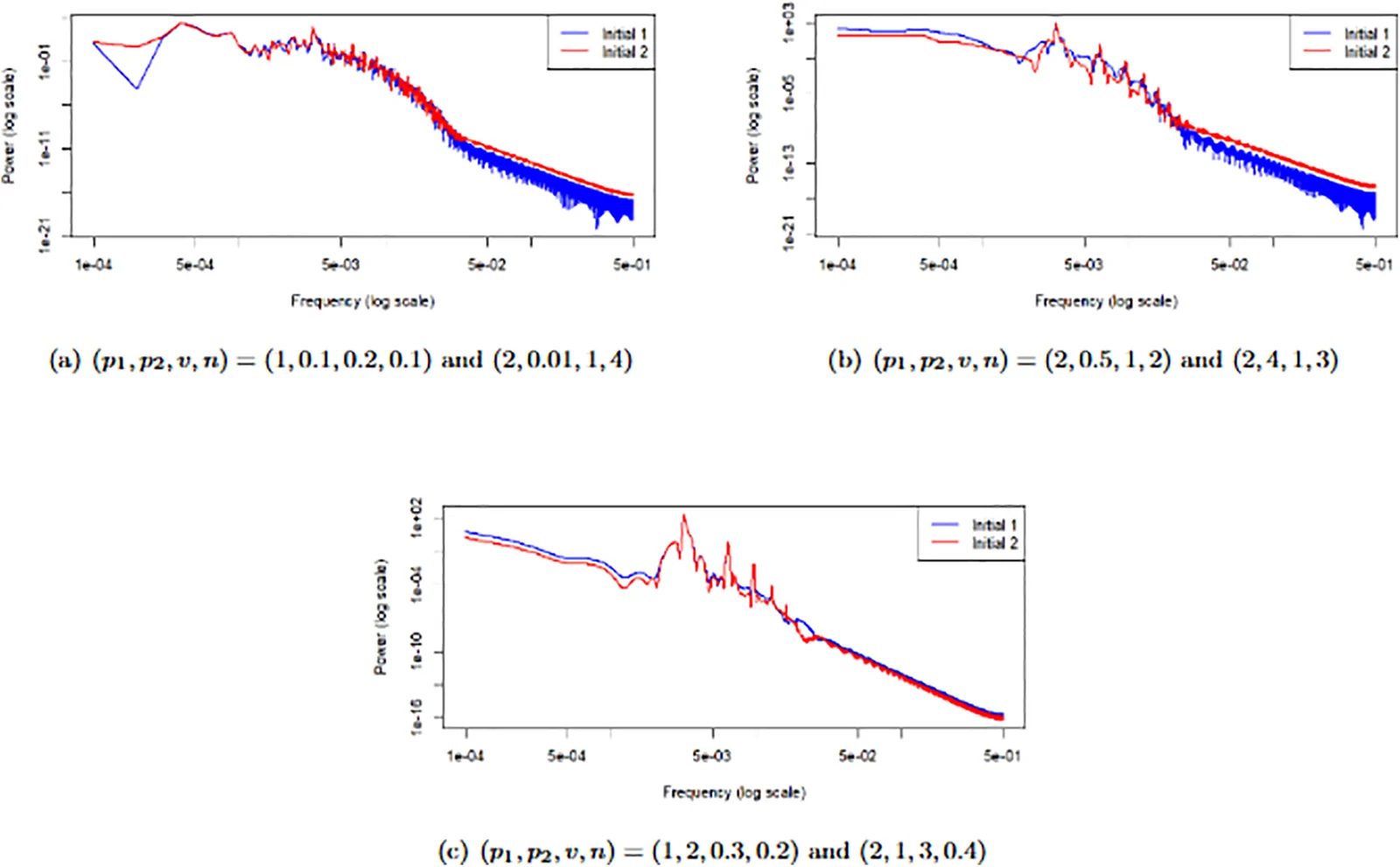
Power spectrum of dynamical system (5) for different parameter sets.

The differences in the spectra of the two initial conditions increase in Fig 9c and the spectrum is spread out over a wider interval in frequency. This means that the oscillatory state is more sensitive to the initial condition. The parameters *q* and *G* increase nonlinear frequency coupling, and the parameter *m* changes the intrinsic time scale of the response. The external time scale is given by the forcing frequency ω which can interact with the intrinsic frequencies and give rise to frequency competition and the loss of simple frequency locking. The topological charge *Q* also changes the effective nonlinear dynamics and thus affects the stability of the resulting oscillatory state. Therefore, the variations in the spectrum shown in Fig 9 (a)−(c) are not just the appearance of the curves, but also the changes in their frequency contents due to the changes in parameters. Periodic motion is associated with a single discrete frequency, multiple discrete frequencies are evidence of quasi-periodic motion, and a broad continuous spectrum accompanied by the phase space and dynamical diagnostics is evidence of chaotic dynamics.

### Return map

Return maps are complementary representations of the dynamics of the system because they correlate *p*_1_(*T*) to the previous state $p_{1}(T+\tau )$. The delay τ is important because it specifies the time scale on which the evolution is sampled, and thus highlights other aspects of the evolution. As shown in Fig 10a, with τ=1 the return points don’t spread away from an identifiable return relation between the successive states.With τ=1 the return points are still concentrated around an identifiable return relation between successive states as shown in Fig 10a. This structure of the trajectory suggests that there is a very strong correlation over the chosen time interval and that the trajectory motion is regular and periodic. The regularity corresponds to the ratio of the dynamics of the system itself and the parameter-dependent nonlinear terms.

**Fig 10:**
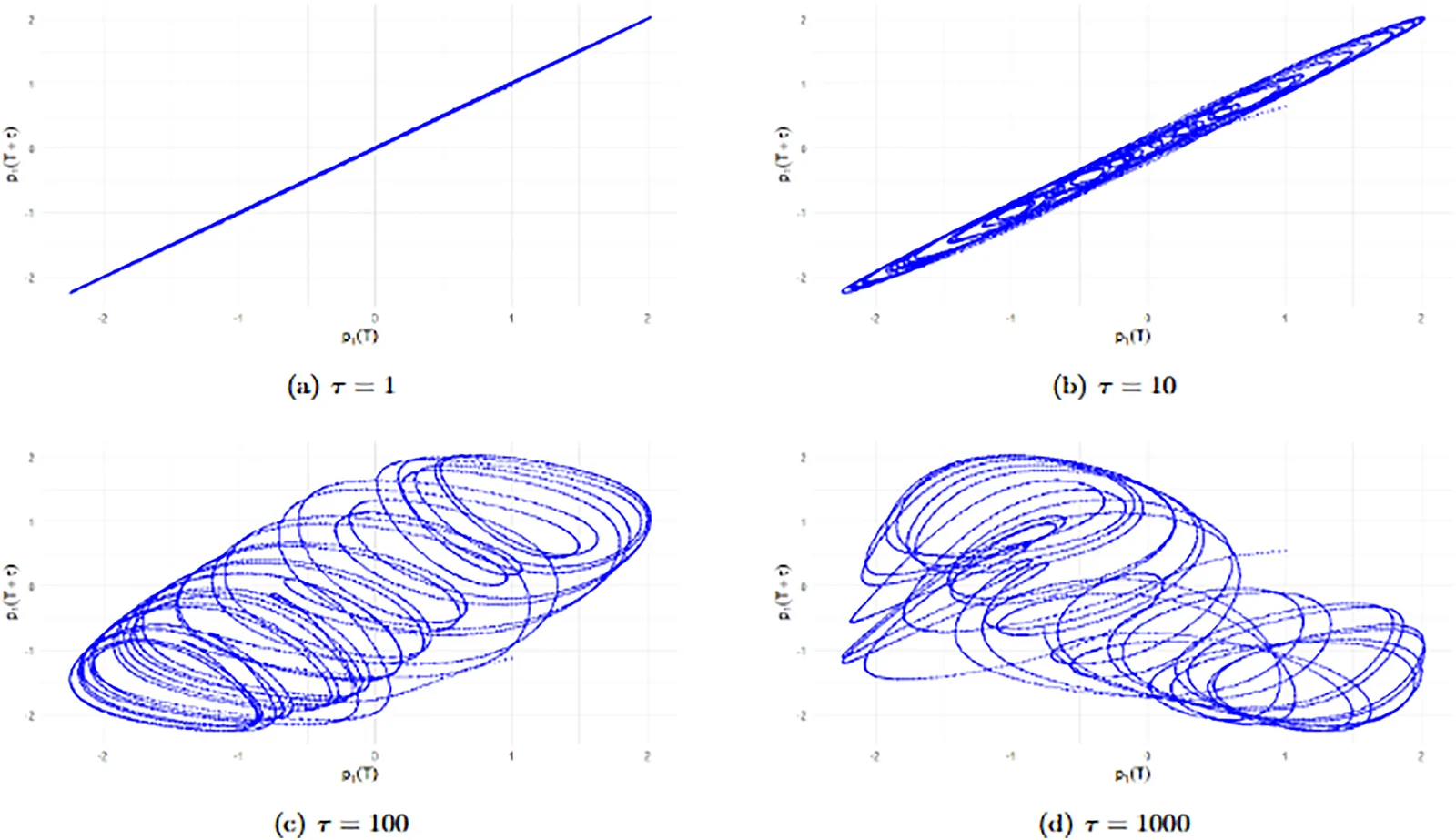
Return map of the dynamical system (5) for (*G*, *q*, *m*, *k*, *l*, *Q*, *w*) = (0.25,0.05,0.1,0.3,0.8,4,2) for initial condition $\left(p_{1},p_{2},v,n)=(1,0.1,0.2,0.1\right)$.

At τ=10 in Fig 10b the return relation is no longer confined to a limited neighborhood and other branches emerge. This means that a different dominant phase relation is now in effect in the sampled state after the delay. This behavior can be caused by the interplay of the intrinsic oscillation and the characteristic forcing frequency ω; on the other hand, the nonlinear parameters *q* and *G* affect the generation of other dynamical components. The resulting structure is therefore consistent with quasi-periodic dynamics. When the return point are plotted as in the Fig 10c with τ=100 the return points are in a much wider area of the map. This indicates a higher degree of loss of short-time correlation between *p*_1_(*T*) and $p_{1}(T+\tau )$. The nonlinear coupling that is controlled by *q* and *G* in addition to the time-scale modification that is controlled by *m* can lead to increased mixing of the frequencies and less coherence in the trajectory. The resulting distributed structure suggests a shift to more irregular dynamics.

Fig 10d shows the return map for τ=1000 where the structure is very spread out and no single, unambiguous relation exists between the successive points in the delayed state. This behavior means that there is significant time memory and increased sensitivity to the previous position. The topological charge *Q* changes the effective nonlinear dynamics, the balance of external forcing, coupling, nonlinear interaction and intrinsic time scales, being controlled by ω,G,q and *m*. These effects can combine to cause the system to move away from regular frequency-locked motion into aperiodic motion. Hence, the changes from an organized return relation at short values of τ to a distributed structure at longer τ suggest an increasing dynamical complexity over the range of sampled time scales. The map shown must however be consistent with the corresponding power spectrum, phase-space structure, and other dynamical diagnostics in order to be considered a chaotic regime.

### Noise detection map

The stochastic perturbations and the detection of noisy regions of the system is shown in Fig 11. The fluctuations in *p*_1_(*T*) shown in Fig 11a suggest that the behavior of the system is altered from the regular oscillatory response by the external perturbation. The effect is not a linear temporal response, implying that the nonlinear dynamics play a key role in the magnitude of the response to the perturbation. Fig 11b shows the time intervals where the deviation from the regular dynamics is above the noise detection criterion chosen. The irregular occurrence of these regions suggests that the system is not equally sensitive to stochastic perturbations. Instead, it’s sensitivity is determined by the immediate dynamical state. This behavior depends on the ratio of the external forcing frequency ω with respect to the nonlinear terms set by *q* and *G* and the intrinsic time scale of *m*.

**Fig 11:**
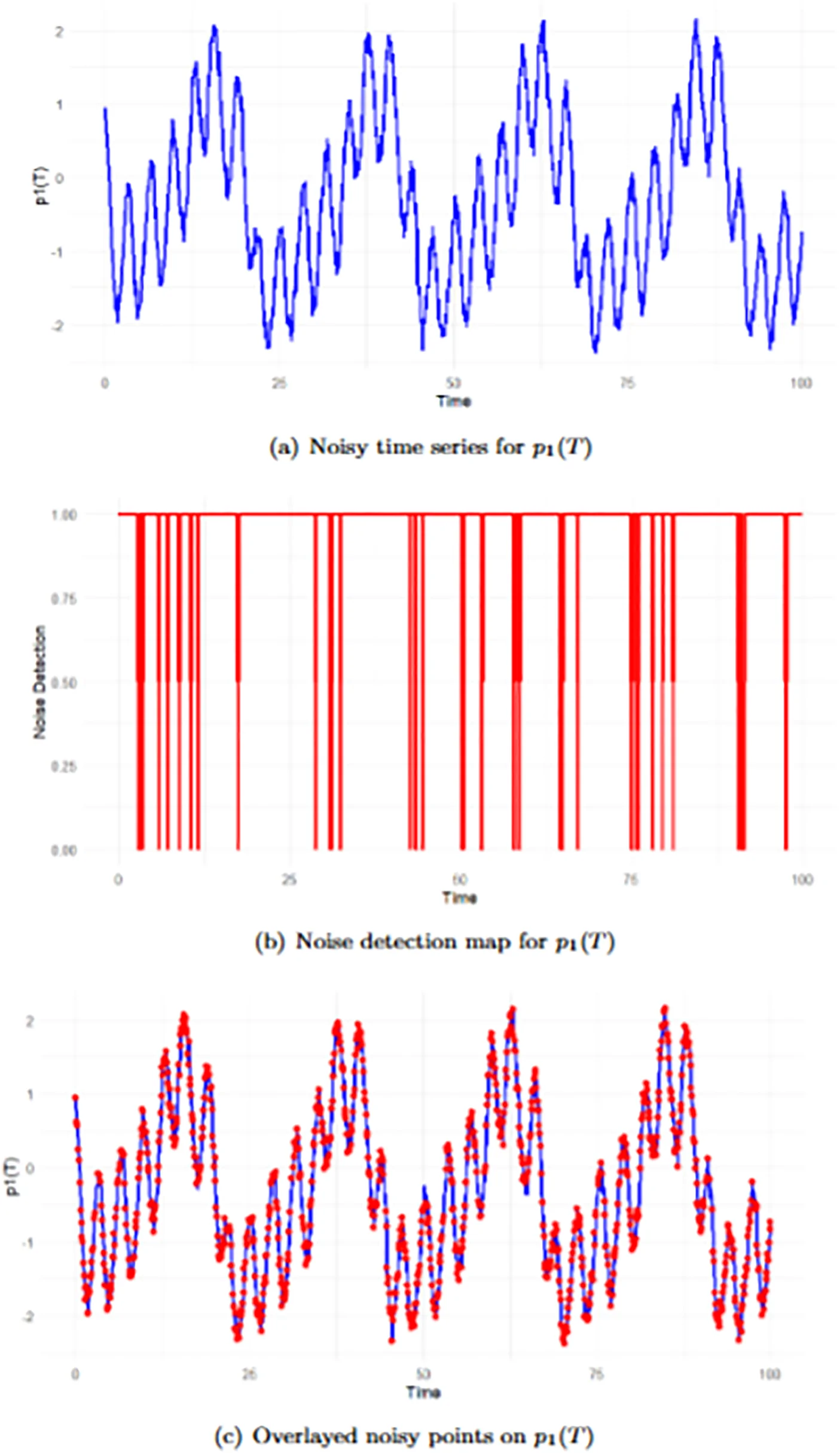
Noise detection map of dynamical system (5) for (*G*, *q*, *m*, *k*, *l*, *Q*, *w*) = (0.25,0.05,0.1,0.3,0.8,4,2) for initial condition $\left(p_{1},p_{2},v,n)=(1,0.1,0.2,0.1\right)$.

The effective nonlinear term is also affected by the topological charge *Q* which may affect the stability of the underlying oscillatory state. As a result, parameter changes may effect the amplitude of the response to noise as well as the length of the periods of time that are detected to be affected by noise. The temporal correlation of the perturbation in the original signal with the detected regions can be seen in Fig 11c which is a direct indication of when the stochastic component significantly influences the system response. The results indicate that the fluctuations seen are not simply considered to be irregularities in the visual system, but rather an aspect of the sensitivity of the nonlinear system to the stochastic perturbations used in the calculations. The degree of deviation from the regular response in the underlying response is associated with reduced dynamical robustness in regions with strong deviations, and high dynamical robustness in regions with weak deviations.

### Disturbance plot

The behavior of *p*_1_ under an external disturbance on *T* = 20 is presented in Fig 12. Prior to the disturbance, *p*_1_ is in a steady, periodic regime, suggesting that the system is operating in a stable dynamical regime. For *T* = 20 the perturbation causes a sudden shift in the trajectory followed, followed by a modified oscillatory response. Such transients are a consequence of the interplay between the disturbance imposed and the natural restoring and nonlinear mechanisms of the system. After a time *T* = 20 the answer depends on the balance of the dynamics, which is determined by the parameter. *G* is the coupling parameter and it changes the interaction between the dynamical variables, and hence the way in which the perturbation is transferred across the system.

**Fig 12:**
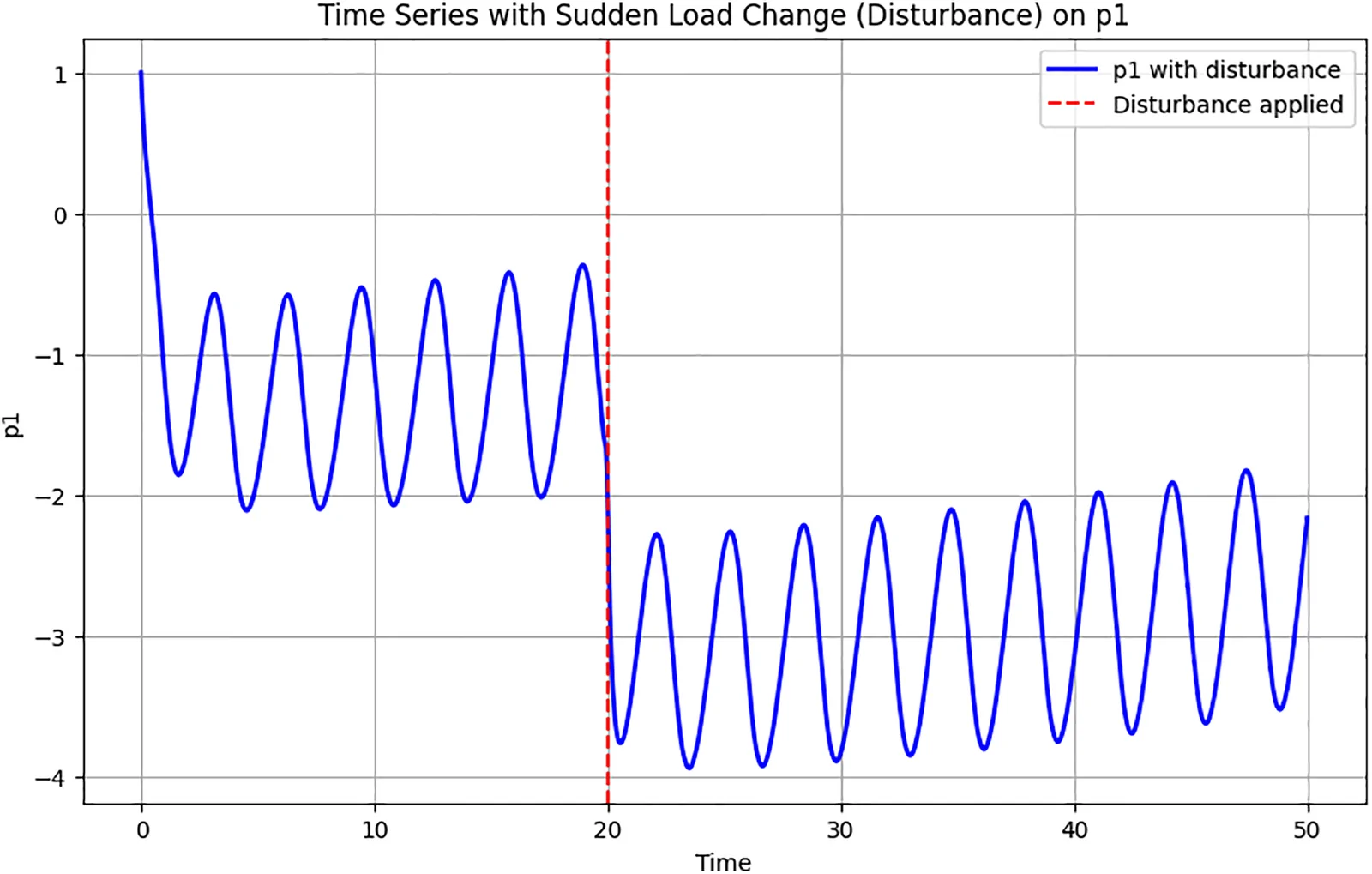
Disturbance plot of dynamical system (5) for (*G*, *q*, *m*, *k*, *l*, *Q*, *w*) = (0.25, 0.05, 0.1, 0.3, 0.8, 4, 2) for initial condition $\left(p_{1},p_{2},v,n\right)$ = (1, 0.1, 0.2, 0.1).

The nonlinear parameter *q* controls the strength of the nonlinear response and the recovery of the oscillatory state is affected by the modification of the characteristic time scale, namely by the parameter *m*. The forcing frequency ω is what controls the time scale of the external forcing and can affect the amplitude and duration of the post-disturbance oscillations. The effective nonlinear contribution is modified by the topological charge *Q* and this can lead to a change in the stability of the perturbed state. The change after *T* = 20 is thus not a mere downwards change in *p*_1_ but rather the transient response of the coupled nonlinear system to the sudden perturbation. The following oscillations show the dynamics of the system as it responds to Q,ω,G,q and *m*. This interpretation further separates the transient from an intrinsic chaotic dynamics that cannot be confirmed by the phase space and spectral analysis.

### Attractors

Attractors give a representation of phase space of the long-term states of evolution to which the system solutions tend. The geometry of the attractor depends on the relative strengths of the nonlinear interaction, coupling, external forcing and internal time scales of the system. In order to interpret the changes in Fig 13 terms of the dynamics rather than just the visual complexity of the trajectories, the parameter-dependent evolution of the dynamics is considered. In Fig 13a the trajectory is confined and is a well organized series of loops. The multiple but not exactly the same excursions are present suggesting that there is more than one characteristic frequency present in the dynamics, a feature that would be consistent with quasi-periodic dynamics. Simple frequency locking is prevented by the interaction between the forcing frequency ω and the intrinsic frequencies of the system and this multi-frequency response arises.

**Fig 13:**
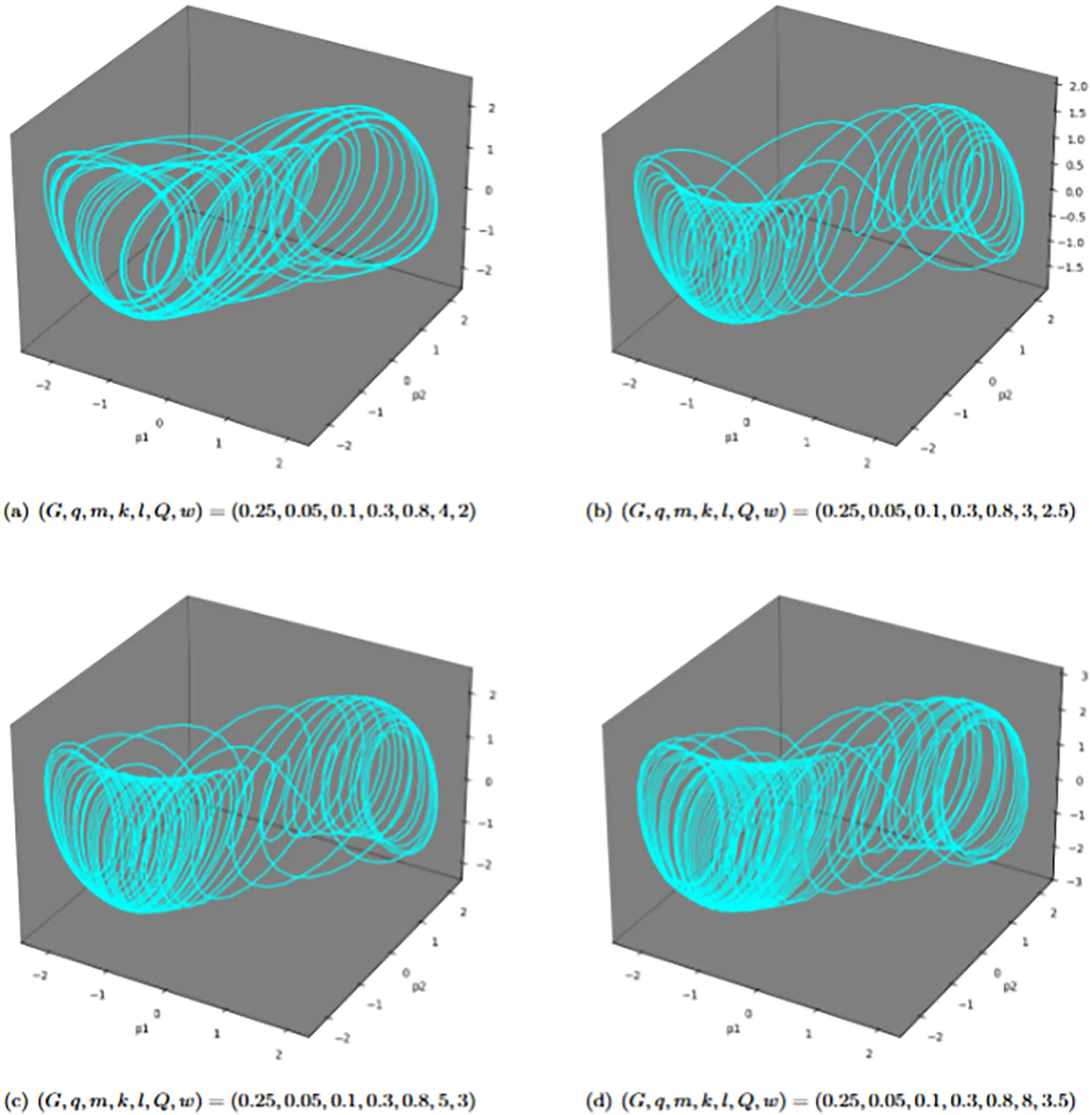
Attractors graphs for dynamical system (5) with initial condition $\left(p_{1},p_{2},v,n)=(1,0.1,0.2,0.1\right)$.

The higher the overlap of the trajectory the more interaction between the dynamical variable is found in Fig 13b. This interaction can be altered by the coupling parameter *G* and the nonlinear parameter *q* alters the deviation from the phase space trajectory. As a result, the system goes further away from the single closed orbit without leaving the bounded area, suggesting better quasi-periodic behavior than a purely periodic state. In Fig 13c, the trajectory is confined to several different regions in the phase space but not to a single closed orbit. The presence of characteristic time scales in the response suggests this structure. The parameter *m* affects the intrinsic time scale of the system while the parameter ω affects the external time scale; their interaction is able to give rise to additional frequency components and maintain the bounded multi-loop structure. In the phase space picture in Fig 13d, the phase space trajectory is denser, which means that the mixing of the dynamical components is stronger. The nonlinear coupling can be improved by the combined effects of *G* and *q* and the relative time scales of the interacting modes can be changed by the change associated with *m* and ω. The topological charge *Q* also changes the effective nonlinear dynamics and is potentially capable of affecting the stability and geometry of the attractor. Overall, a multi-frequency response becomes more organized in Fig 13 (a)−(c), leading to increasingly complex bounded dynamics as the relevant parameterFIs change the balance between the forcing, coupling, nonlinearity and intrinsic time scales.

### Bifurcation analysis

The bifurcation diagram in Fig 14 shows the response of the periodically forced nonlinear circuit as a function of the forcing amplitude *Q*, where the system state is sampled once per forcing period using a stroboscopic (Poincare) sampling procedure. For Q≲2.8, the sampled points form a broad and densely distributed band, indicating that the response does not converge to a single fixed stroboscopic value and is consistent with quasi-periodic dynamics associated with motion on a torus. As the forcing amplitude approaches Q≈2.8, the organization of the stroboscopic response changes markedly, indicating a transition in the underlying dynamical regime. For Q≳2.8, the scattered band collapses into a much narrower and more organized branch, indicating that the system becomes phase-locked to the external forcing and approaches a more regular periodic response. Thus, the bifurcation diagram provides evidence of a qualitative transition from a quasi-periodic regime at lower forcing amplitudes to a more regular periodic regime at higher forcing amplitudes.

**Fig 14:**
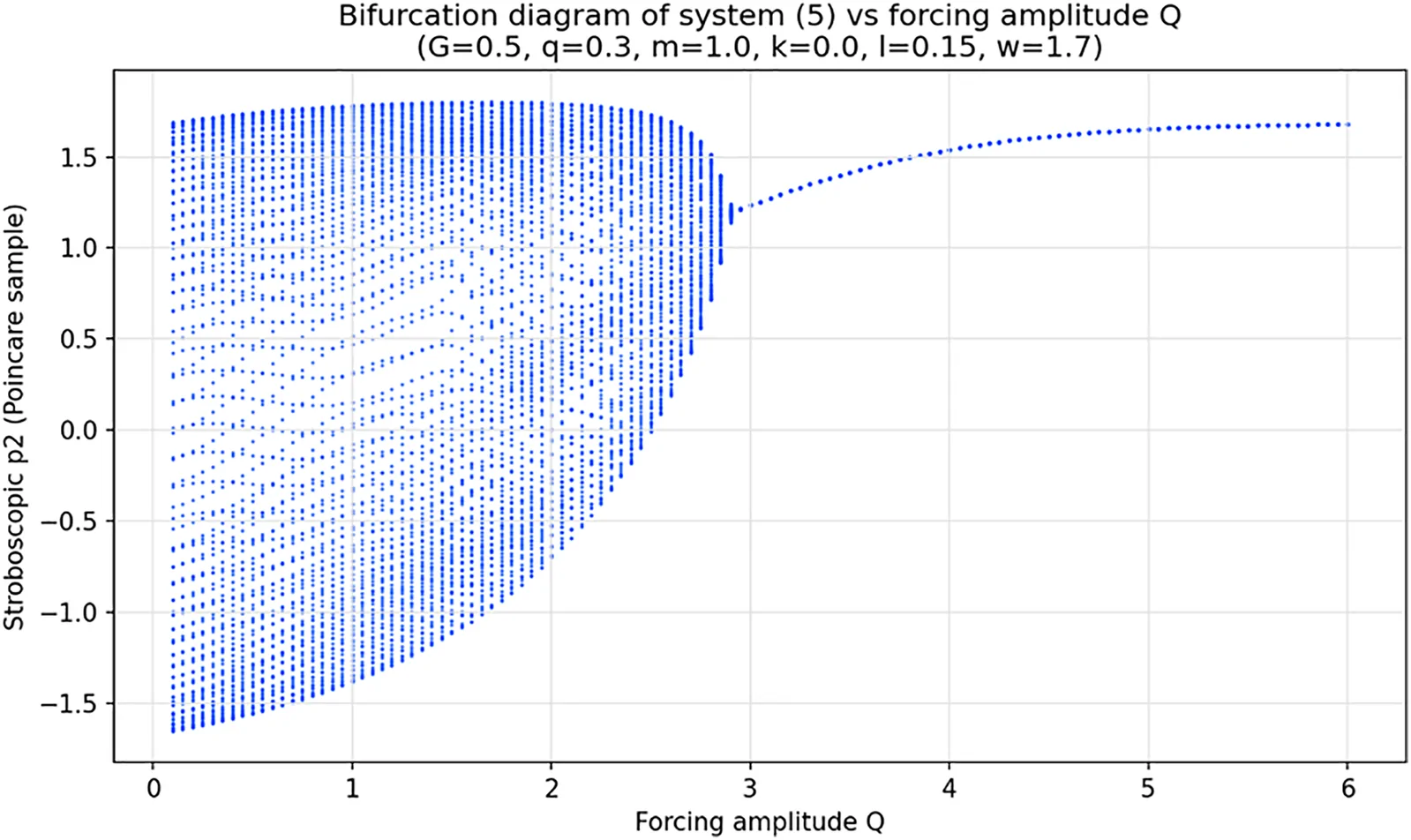
Bifurcation diagram versus forcing amplitude *Q.* The band of scattered points below Q≈2.8 corresponds to the quasi-periodic (torus) regime; above this threshold, the system locks onto a single periodic orbit, and the stroboscopic points collapse onto a smooth curve.

### Lyapunov Exponents

In Fig 15 the Lyapunov-exponent spectrum $\lambda_{1},\lambda_{2},\lambda_{3}$ is shown as a function of the forcing amplitude *Q*. The largest Lyapunov exponent $\lambda_{1}$ is the main measure of exponential sensitivity to initial conditions and positive values are an indication of chaotic dynamics. In the whole range of the investigated *Q*, no chaotic dynamics is found, as $\lambda_{1}$ is always non-positive, and it is concluded that there is no evidence of chaotic dynamics in the range of the considered *Q*. If *Q* < 2.8 then $\lambda_{1}$ will be very close to zero. This neutral-exponent structure, along with the approximately zero fourth exponent in the plot, is consistent with quasi-periodic motion on a two-torus in the periodically forced formulation, and agrees with the wide band of stroboscopic points seen in the bifurcation diagram. The largest Lyapunov exponent shifts from a neutral value towards negative values as *Q* approaches 2.8 where the qualitative change in the bifurcation diagram is noticed. When Q≳2.8 $\lambda_{1}$ is clearly negative and the other non-neutral exponents are also negative, thus indicating a stable, phase-locked periodic response. In Fig 16, to investigate the running Lyapunov-exponent estimates, we look at the convergence of these estimates for integration time for two different values of *Q*: *Q* = 1.0 for the quasi-periodic regime and *Q* = 5.0 for the phase-locked regime. In both cases, the values of the running estimates start off differently due to the transient dynamics and converge to more or less constant values, showing the convergence of the finite-time Lyapunov estimates. If *Q* = 1.0 then the largest exponent tends to zero, as expected following a stable quasi-periodic state, but if *Q* = 5.0 then the largest exponent tends to a definite negative value, as expected following a stable phase-locked periodic state. Hence, the Lyapunov-exponent analysis supports the transition found in the bifurcation diagram, from the quasi-periodic regime at lower values of the forcing amplitude to the regular regime of phase locking at higher values of the forcing amplitude, without any evidence for a transition to chaos domain in the explored forcing amplitude range.

**Fig 15:**
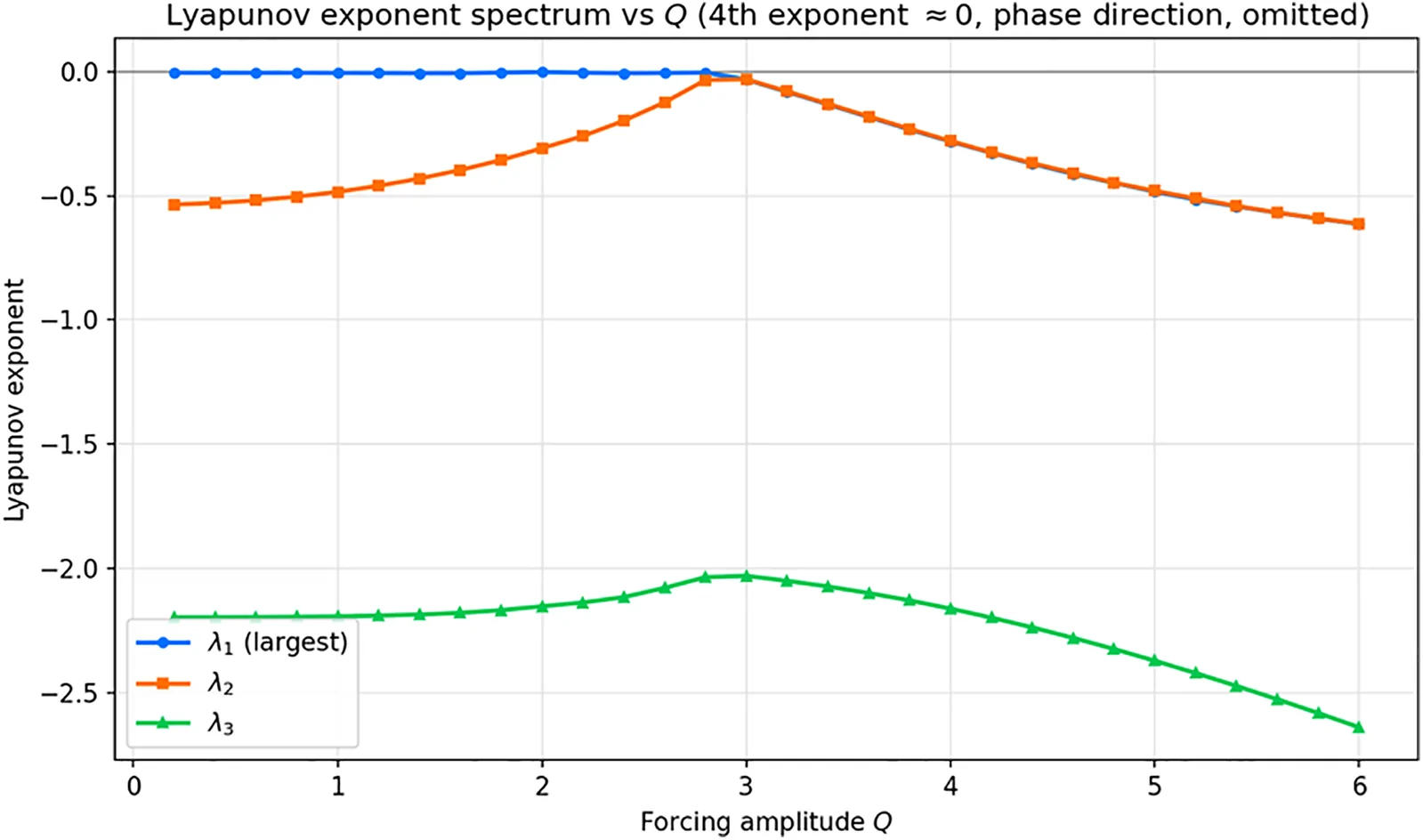
Figure shows $\lambda_{1},\lambda_{2},\lambda_{3}$ versus *Q.* The largest exponent $\lambda_{1}$ remains near zero in the quasi-periodic regime, consistent with a two-torus, and becomes negative near Q≈2.8, indicating phase locking in agreement with the bifurcation diagram. No exponent becomes positive, confirming the absence of chaos.

**Fig 16:**
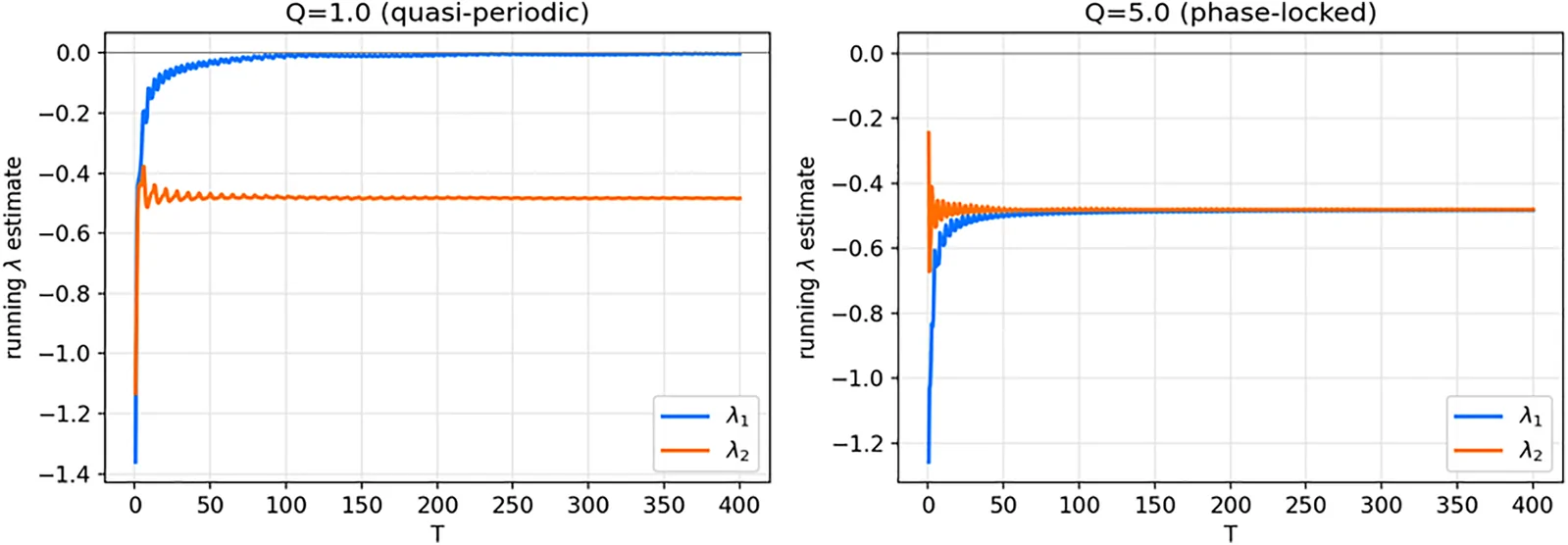
Convergence of the running Lyapunov-exponent estimate for representative quasi-periodic (*Q* = 1.0) and phase-locked (*Q* = 5.0) cases, confirming convergence.

### Poincare maps

In Fig 17 as the amplitude of the forcing *Q* is increased, the dynamical behavior of the system clearly changes. When *Q* = 0.5,1.5 and 2.6, the stroboscopic points lie on smooth closed invariant curves that suggest quasiperiodic dynamics on an invariant torus. Even though the geometry of these curves does vary with *Q* the structure of the curves is kept organized, hence the system is still quasiperiodic in this parameter range. For *Q* = 3.2 and 4.0 and 5.5 however, the Poincare points reduce to effectively a single stroboscopic state with very small residual variations of order 10^–8^−10^–11^. This collapse suggests that the system will be synchronized to the external periodic forcing, as is the case for a phase-locked period-1 orbit for which $x(t+T_{f})\approx x(t)$. In this way, the Poincare sections indicate the presence of a quasiperiodic-to-periodic phase-locked transition between *Q* = 2.6 and *Q* = 3.2. The extremely small apparent spreads in the higher-*Q* panels should not be interpreted as distinct attractor structures, but rather as numerical-level deviations around the phase-locked state. Together with the non-positive largest Lyapunov exponent and the absence of a diffuse or fractal Poincare structure, these results provide consistent evidence for regular, quasiperiodic, and periodic dynamics rather than deterministic chaos over the investigated range of *Q*.

**Fig 17:**
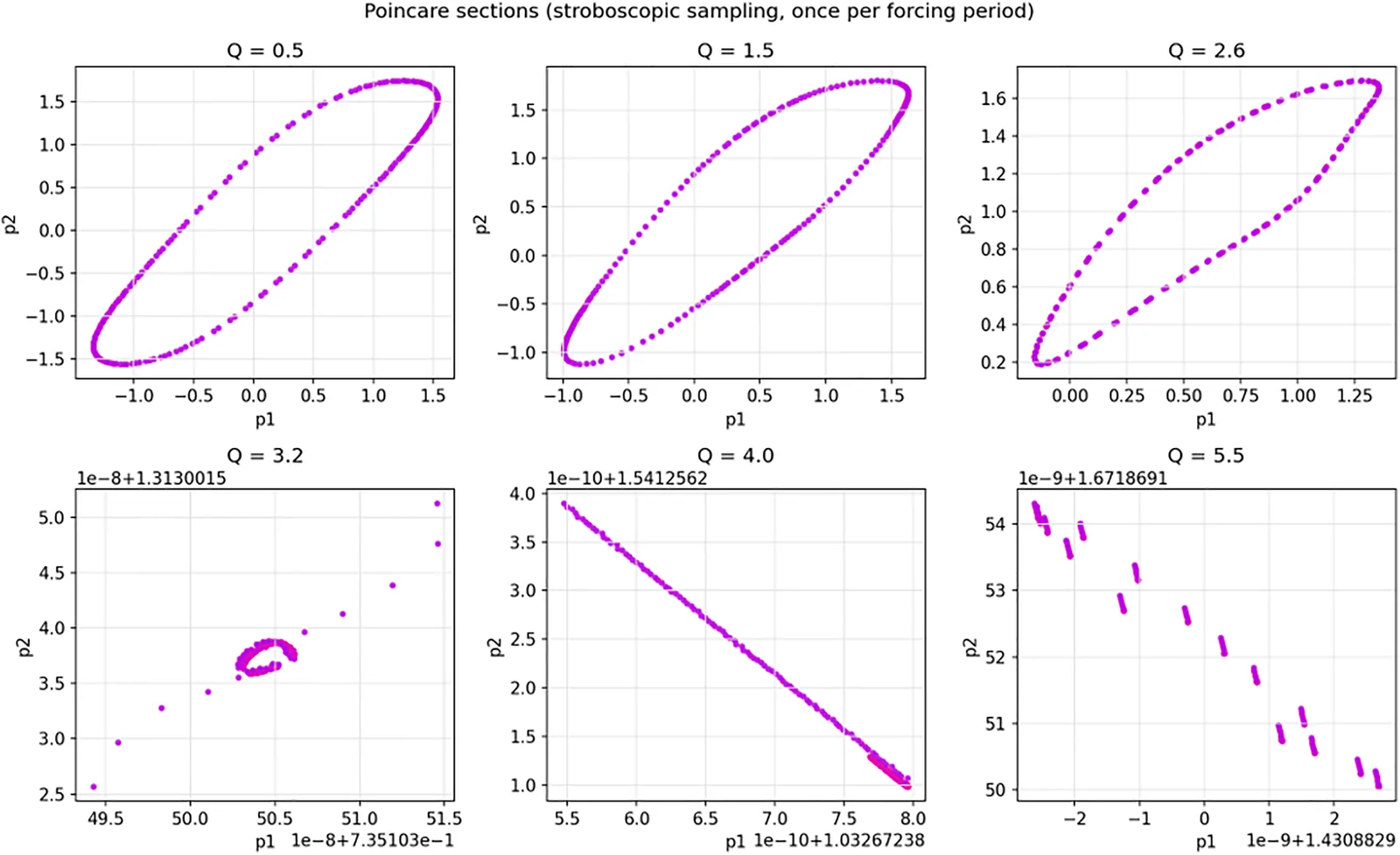
Poincare maps for different forcing amplitudes *Q.* Closed invariant curves for *Q* = 0.5, 1.5, and 2.6 indicate quasiperiodic dynamics, whereas the collapse of the stroboscopic points for *Q* = 3.2, 4.0, and 5.5 indicates phase-locked periodic behavior.

## Conclusions

The study reveals that neural dynamics are captured by the pressure-sensitive FHN neuron model, which is realized by two capacitors and piezoelectric elements. The addition of a nonlinear function (without a multiplier) also makes it easier to realize the circuit in hardware, making it more suitable for neuromorphic hardware. An exhaustive parameter space study was performed to learn about system behavior across different parameters. The influences of the different values of *G*, *q* and *m* were assessed using statistical methods and eigenvalue analysis. An additional sensitivity analysis was also performed using an ensemble size to assess the robustness of the statistical characterization framework. A comparison of the empirical cumulative distribution function and kernel density estimation as the number of ensembles grew showed that the resulting statistical results were stable and reproducible. The observation of these different dynamical regimes with the same number of sampled initial conditions also gives more confidence to the parameter space classification. The results show that the system is unstable in most parts of the region; diverging trajectories are the main results, and periodic behavior is only found in restricted regions. The study of the eigenvalues also separates three stability regimes. These observations show that the good behavior of the system is highly sensitive to the correct tuning of parameters and the correct initialization, and they also highlight the necessity of using appropriate stabilization strategies. If we now apply an external periodic force to the system, the dynamics become more complicated. Methods such as power spectral density, return maps, attractor reconstruction, noise analysis, and disturbance plots are used to analyze quasi-periodic and chaotic responses. Numerous firing patterns, such as periodic, quasi-periodic, and chaotic firing, have been observed in both simulations and hardware implementation, with good agreement found with biological neuron activity. Overall, the results show the high sensitivity of the system to the circuit parameters, including capacitance, resistance, and inductance, which directly affect the oscillation, damping, and stability of the system. The controllability allows fine tuning of neural-like responses. The analysis is useful for the design of neuromorphic computing systems, neural prosthetics, and brain-machine interfaces, and it provides a foundation for modeling and implementing bio-inspired models of neural dynamics.
